# Longitudinal multi-omics of host–microbe dynamics in prediabetes

**DOI:** 10.1038/s41586-019-1236-x

**Published:** 2019-05-29

**Authors:** Wenyu Zhou, M. Reza Sailani, Kévin Contrepois, Yanjiao Zhou, Sara Ahadi, Shana R. Leopold, Martin J. Zhang, Varsha Rao, Monika Avina, Tejaswini Mishra, Jethro Johnson, Brittany Lee-McMullen, Songjie Chen, Ahmed A. Metwally, Thi Dong Binh Tran, Hoan Nguyen, Xin Zhou, Brandon Albright, Bo-Young Hong, Lauren Petersen, Eddy Bautista, Blake Hanson, Lei Chen, Daniel Spakowicz, Amir Bahmani, Denis Salins, Benjamin Leopold, Melanie Ashland, Orit Dagan-Rosenfeld, Shannon Rego, Patricia Limcaoco, Elizabeth Colbert, Candice Allister, Dalia Perelman, Colleen Craig, Eric Wei, Hassan Chaib, Daniel Hornburg, Jessilyn Dunn, Liang Liang, Sophia Miryam Schüssler-Fiorenza Rose, Kim Kukurba, Brian Piening, Hannes Rost, David Tse, Tracey McLaughlin, Erica Sodergren, George M. Weinstock, Michael Snyder

**Affiliations:** 1 Department of Genetics, Stanford University School of Medicine, Stanford, CA USA; 2 The Jackson Laboratory for Genomic Medicine, Farmington, CT USA; 3 Department of Medicine, UConn Health, Farmington, CT USA; 4 Department of Electrical Engineering, Stanford University, Stanford, CA USA; 5 Stanford Center for Genomics and Personalized Medicine, Stanford, CA USA; 6 Division of Endocrinology, Stanford University School of Medicine, Stanford, CA USA; 7 Stanford Diabetes Research Center, Stanford, CA USA; 8 Spinal Cord Injury Service, Veteran Affairs Palo Alto Health Care System, Palo Alto, CA USA; 9 Department of Neurosurgery, Stanford University School of Medicine, Stanford, CA USA; 10 Earle A Chiles Research Institute, Providence Cancer Center, Portland, OR USA; 11 Donnelly Centre for Cellular & Biomolecular Research, University of Toronto, Toronto, Ontario Canada

**Keywords:** Pre-diabetes, Prognostic markers

## Abstract

Type 2 diabetes mellitus (T2D) is a growing health problem, but little is known about its early disease stages, its effects on biological processes or the transition to clinical T2D. To understand the earliest stages of T2D better, we obtained samples from 106 healthy individuals and individuals with prediabetes over approximately four years and performed deep profiling of transcriptomes, metabolomes, cytokines, and proteomes, as well as changes in the microbiome. This rich longitudinal data set revealed many insights: first, healthy profiles are distinct among individuals while displaying diverse patterns of intra- and/or inter-personal variability. Second, extensive host and microbial changes occur during respiratory viral infections and immunization, and immunization triggers potentially protective responses that are distinct from responses to respiratory viral infections. Moreover, during respiratory viral infections, insulin-resistant participants respond differently than insulin-sensitive participants. Third, global co-association analyses among the thousands of profiled molecules reveal specific host–microbe interactions that differ between insulin-resistant and insulin-sensitive individuals. Last, we identified early personal molecular signatures in one individual that preceded the onset of T2D, including the inflammation markers interleukin-1 receptor agonist (IL-1RA) and high-sensitivity C-reactive protein (CRP) paired with xenobiotic-induced immune signalling. Our study reveals insights into pathways and responses that differ between glucose-dysregulated and healthy individuals during health and disease and provides an open-access data resource to enable further research into healthy, prediabetic and T2D states.

## Abstract (web summary)

Deep profiling of transcriptomes, metabolomes, cytokines, and proteomes, alongside changes in the microbiome, in samples from individuals with and without prediabetes reveal insights into inter-individual variability and associations between changes in the microbiome and other factors.

## Main

T2D is a metabolic disorder that affects more than 400 million people worldwide^1^. Prediabetes, or intermediate hyperglycaemia, represents a high-risk state for developing T2D that is often undiagnosed. Approximately 5–10% of people with prediabetes will become diabetic each year, and up to 70% of individuals with prediabetes will eventually develop diabetes in their lifetime. Prediabetes and T2D are often associated with insulin resistance, where individuals produce insulin but are hyperglycaemic because their cells do not respond to insulin. Studying individuals with prediabetes and insulin resistance offers unique opportunities to investigate the earliest stages of diabetes, including its effect on biological processes and health.

The development of T2D involves complex and heterogeneous processes^2,3^, and individuals with diabetes show differences in both human host markers and gut microbiome signatures when compared with healthy individuals^4,5^. Furthermore, physiological stresses, such as viral infection, have been linked to the development of diabetes^6–8^. However, a global and simultaneous profile of both host and microbial molecules in prediabetes is lacking. We still do not have a systematic understanding of how healthy individuals and those with prediabetes differ at baseline or change over time or in response to stresses such as viral infections, and whether such responses depend on insulin-resistance status.

To obtain a better understanding of the biological processes associated with both healthy individuals and those with T2D at its earliest stages, we performed a detailed longitudinal analysis of individuals with various early impairments in glucose control (for example, insulin resistance). We carried out deep profiling of the human gut and nasal microbiomes, as well as host circulating blood during periods of health and stress. Our study reveals that many host biochemical and microbial components are stable over time when healthy, but can undergo dynamic and marked changes in response to viral infection and other perturbations. Notably, these changes differ between insulin-sensitive and insulin-resistant individuals. As well as revealing biological insights, our study provides a rich, open-access resource of longitudinal data that allows deep mining of host and microbial changes at an individual level.

## Overview: cohort, sample collection and assays

**Fig. 1:**
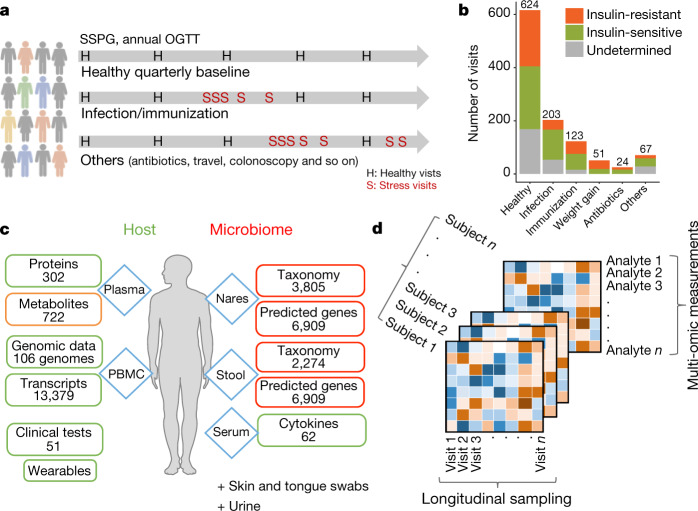
Summary of study design, cohort details and data. **a**, Samples were collected from 106 participants for nearly four years, with quarterly healthy visits and dense sampling during stress events. **b**, Summary of visits in each category and categorization of participants as insulin-sensitive or insulin-resistant. **c**, Sample sources, types of multi-omic assay and number for each data type profiled per visit. **d**, Conceptual illustration of the data structure for downstream analysis. Supplementary Table 3 lists the number of visits and assays profiled for each participant.

We followed 106 participants for up to almost four years (Fig. 1a; Stanford IRB No. 23602), except for one individual who was followed for seven years; early findings for this individual have been published previously^8^. The cohort comprised 55 women and 51 men, with ages ranging from 25 to 75 years old and body mass indexes (BMI) ranging from 19 to 41 kg m^–2^ (Supplementary Table 1).

**Extended Data Fig. 1:**
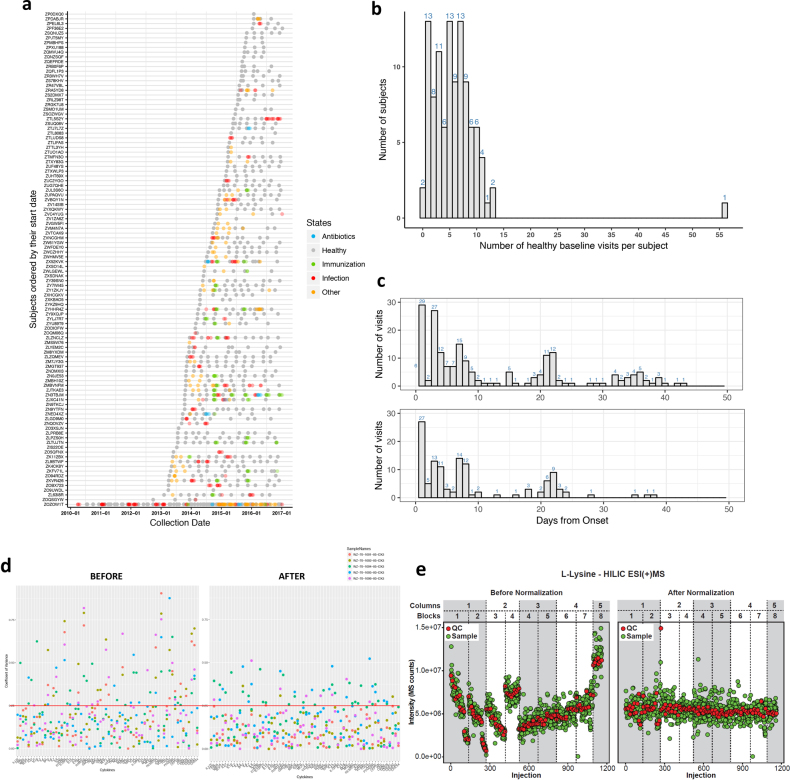
Summary of visits sampled and technical normalization across the study. **a**, Visit chart along the time axis illustrated for each subject. **b**, Number of healthy baselines across the study per subject. **c**, Number of event visits (top, RVI; bottom, immunization) since the onset of events. Visits with defined days from onset of events were counted. **d**, Comparison of before and after normalization of cytokine data in order to correct batch effects during the assay. The after-normalization data show overall reduced coefficient of variation (CV). Red line, 25% CV. An example is shown for correcting five samples from two batches. **e**, Comparison of before (left) and after (right) LOESS normalization of metabolite data using l-lysine as an example. l-Lysine signal drift with time was independently corrected by fitting a LOESS curve to the l-lysine signal measured in QCs. QCs were injected every ten biological samples and consisted of an equimolar mixture of 150 random samples from the study. Red, l-lysine signals from QCs; green, biological samples in HILIC ESI(+) MS mode.

Samples were generally taken every three months when participants were self-reported as healthy. In total, 624 healthy baselines were profiled, with each participant having 1–56 healthy visits during the study (Fig. 1b, Extended Data Fig. 1a, b). Additional visits during periods of environmental or medical stress included events of respiratory viral infection (RVI; 54 episodes in 32 participants with a total of 203 visits) and immunization (29 episodes in 22 participants with a total of 123 visits) with dense sampling in the early phase (two time points during days 1–6), a later phase (day 7–14) and the recovery phase (at weeks 3 and 5; Fig. 1a, Extended Data Fig. 1c). Samples were also taken when other stresses occurred, such as weight gain, antibiotic treatment, colonoscopy, travel and other self-reported acute severe stresses, but these were less frequent (Fig. 1b). In total, we profiled 1,092 time points across all participants, with a median of seven visits (including a median of five healthy baseline visits) spanning a median of 1.6 years per participant (Supplementary Table 1).

At each visit, blood was collected for host molecular ’omics profiling and two types of sample (stool and nasal swab) were collected for microbial profiling^9^. A battery of molecular and clinical laboratory tests were performed and complemented with self-reported online surveys, which documented changes in medication, physical activity, diet preference and perceived stress level (Fig. 1c). Blood was fractionated into peripheral blood monocytes (PBMCs), plasma and serum. Each participant’s exome was sequenced once; otherwise, for each visit, we profiled 13,379 transcripts (using Ribo-minus RNA sequencing (RNA-seq)) from PBMCs, 722 metabolites (using untargeted liquid chromatography with tandem mass spectrometry (LC–MS/MS)), 302 proteins (using sequential window acquisition of all theoretical mass spectra (SWATH-MS)) from plasma, and 62 cytokines and growth factors from serum. In addition, thousands of gut and nasal microbial taxa and predicted genes were profiled using 16S sequencing (for quality controls, see Methods and Extended Data Fig. 1d, e; Supplementary Table 4 lists all molecules profiled). Note that all visits were intensively characterized by 51 clinical laboratory tests. Supplementary Table 3 lists all study collections, types of ‘omics’ measurement assayed (referred to here as omes) and metadata; 823 visits were profiled with at least six multi-omic assays. Overall, we generated a rich data set containing millions of molecular and microbial measurements (Fig. 1d). Notably, most (*n* = 83) of the individuals consented to make their data open access, thus providing a valuable resource (available at https://portal.hmpdacc.org/ and http://med.stanford.edu/ipop.html).

Because of the interest in studying prediabetes, insulin resistance and T2D, at each visit we measured haemoglobin A1C (A1C; a measure of average glucose over three months: diabetic ≥ 6.5% > prediabetic ≥ 5.7%) and fasting glucose (diabetic ≥ 126 mg dl^–1^ > prediabetic ≥ 100 mg dl^–1^)^10^; 84 participants underwent an annual oral glucose tolerance test (OGTT: diabetic glucose ≥ 200 mg dl^–1^ > prediabetic ≥ 140 mg dl^–1^ at 2 h, Supplementary Table 2). Upon entering the study, 51 participants had prediabetes and 9 had diabetes according to any of the three criteria above. Sixty-six participants without medical contraindications underwent the insulin suppression test^11^ to assess their insulin resistance or sensitivity through steady-state plasma glucose (SSPG); 31 participants were classified as insulin-sensitive (SSPG < 150 mg dl^–1^) and 35 as insulin-resistant (SSPG ≥ 150 mg dl^–1^) with similar profiles in age, sex and ethnicity (Supplementary Table 1). Although this study focuses on different phenotypes and changes that depend on insulin resistance as defined by SSPG, the comparison of different glucose dysregulation defined by A1C, fasting glucose, OGTT and SSPG and their progression during the study are reported in a companion paper^12^.

## Variation in healthy baseline profiles

**Fig. 2:**
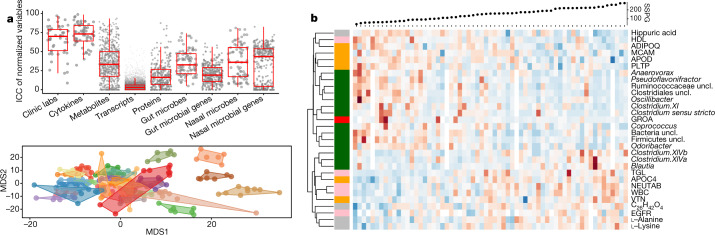
Variances observed among health visits. We characterized 624 healthy baselines. **a**, Top, intra-class correlation (ICC) levels in analytes from each ’ome (gut microbes, median 32.65; gut microbial genes, median 19.19, *P* = 7.261 × 10^−15^ by Wilcoxon-rank test, two-sided). Bottom, separation pattern by multidimensional scaling (MDS) among participants defined by the top 30 most personally distinct analytes. Fourteen participants are shown in different colours, with healthy visits presented as dots bound by contours denoting personal variable space. **b**, Expression of analytes significantly associated with SSPG in healthy baselines. Red, increased expression; blue, decreased expression. Pink, clinical laboratory tests; red, cytokines; grey, metabolites; orange, proteins; dark green, gut microbes at the genus level per row of the heatmap. HDL, high-density lipoprotein; TGL, triglycerides; NEUTAB, neutrophil absolute count; uncl., unclassified; WBC, white blood cell count; EGFR, estimated glomerular filtration rate; GROA, also as CXCL1, growth-regulated alpha protein. Undefined abbreviations are protein symbols.

**Extended Data Fig. 2:**
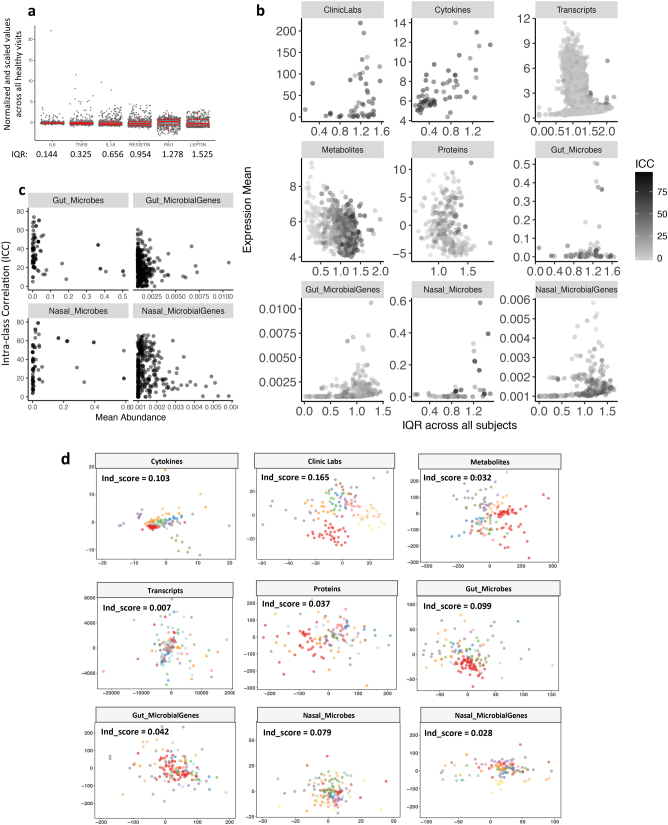
Variance among healthy baselines (***n*** = 624) observed for each measurement. **a**, Examples of measurements with their normalized values and corresponding IQR either outlined by boxes or quantified by numbers below. **b**, Scatter plots show diverse association patterns between IQR and expression or abundance level for measurements across all healthy visits in different ’omes. **c**, Scatter plots of gut and nasal mean abundance versus corresponding ICC for each measurement across all healthy baselines. Each measurement is presented as one grey dot; darkness of grey reflects the degree of overlap of nearby dots. **d**, MDS plots for each ’ome. MDS1 and MDS2 were shown as the *x* and *y* axis, respectively. Only 14 subjects with at least ten healthy baselines were used for plotting to achieve clear visualization of the clustering patterns. Subjects were given the same colour code across plots. Individual separation scores (Ind_score) calculate the average distance between any two individuals across all analytes in each ’ome.

The extensive longitudinal profiling enabled us to assess variation within an individual over time, between individuals, and in different types of molecule and microorganism. We first quantified the dispersion level of multi-omic measurements from all healthy visits across the cohort by examining their interquartile ranges (IQRs). We observed complex and variable patterns of our measurements depending on the ’omic type, analyte expression, and participant structure in our cohort (Extended Data Fig. 2a, b, Supplementary Table 4). We evaluated the proportion of total variance explained by participant structure in the cohort using intra-class correlation (ICC) from linear mixed effect (LME) models in each multi-omic analyte (Fig. 2a, Supplementary Table 5). Clinical laboratory tests (labs) and cytokine profiles were the most personally distinct (for example, more variable between individuals), whereas transcripts had similar variance within individuals as between individuals. Gut microbial taxa, mostly low-abundance ones, showed significantly higher inter-personal variability than their predicted genes (*P* = 7.26 × 10^–15^; Extended Data Fig. 2c). We quantified the separation pattern between participants by individuality scores (ind_score). The bottom panel of Fig. 2a shows the separation pattern between participants based on the top 30 most personally distinct analytes. As more variables were selected, clusters of individuals overlapped more with a decreased individuality score (Supplementary Table 6). Similarly, the degree of separation and individuality score differ among omes depending on the inter-personal variability in analytes from each ome (Extended Data Fig. 2d). We found the highest individual separation (ind_score = 0.165) for clinical labs and the lowest separation (ind_score = 0.007) for transcripts.

**Extended Data Fig. 3:**
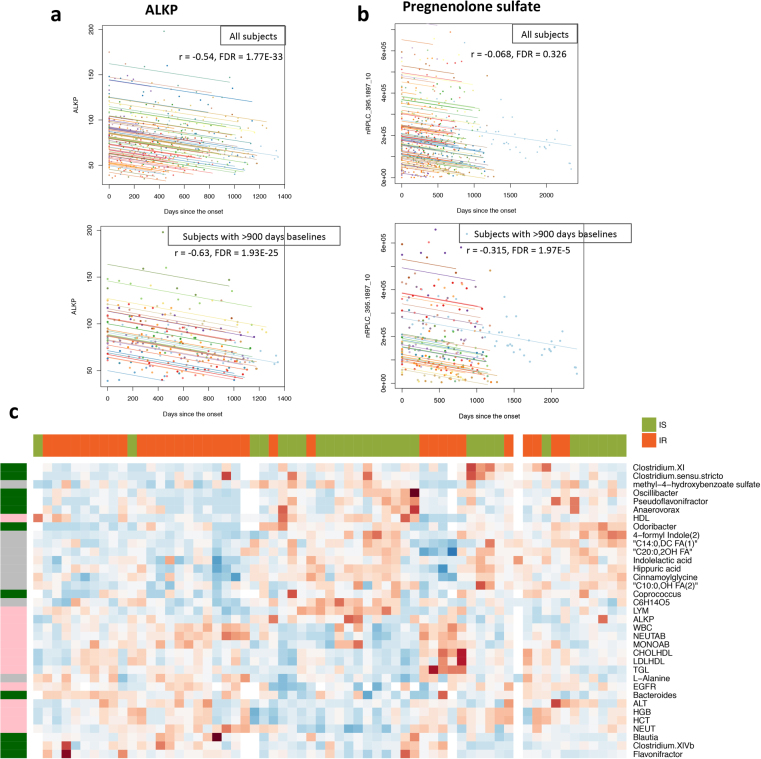
Factors that contributed to the variance among healthy baselines. **a**, **b**, Examples of measurements correlated with the day factor in our study. Rmcorr correlation is used for the association calculations. **a**, The association between the level of ALKP and the day factor was significant both in all 106 subjects and in 27 subjects sampled for more than 900 days. **b**, The association between the level of pregnenolone sulfate and the day factor was significant only in 27 subjects sampled for more than 900 days but not when all 106 subjects were included. Individuals were coloured differently for comparison. **c**, The expression pattern of measurements that differed significantly between insulin-resistant (*n* = 35, red) and insulin-sensitive (*n* = 31, green) subjects in healthy baselines (Wilcoxon-rank test, two-sided). On the left: pink, clinical laboratory tests; grey, metabolites; dark green, gut microorganisms.

We also assessed the influence of time on healthy baseline variation within individuals, with a specific emphasis on markers that increased or decreased monotonically over time. We identified both host molecules and gut microorganisms that significantly correlated, positively or negatively, with time (examples in Extended Data Fig. 3a, b, Supplementary Table 7). Among markers that decreased over time, some have been described previously (for example, alkaline phosphatase, ALKP^13^; *q* = 1.77 × 10^–33^), and some have not, to our knowledge (for example, corpuscular haemoglobin, MCH; *q* = 6.08 × 10^–14^). Several microbiome genera also decreased over time, including Erysipelotrichaceae unclassified, *Butyricicoccus*, and *Akkermansia* (q < 0.05). Expanded ageing-related results will be presented elsewhere (submitted).

## Factors that correlate with insulin resistance

As many of our participants were characterized with respect to insulin resistance, we sought to characterize co-associations using two approaches: regression analysis with SSPG values and co-association with insulin-sensitive and insulin-resistant participants (association of analytes with A1C, high-sensitivity C-reactive protein (HSCRP) and other clinical measurements are described below and in more detail in the companion paper^12^). At constant BMI, SSPG values rarely varied^14^ per participant in our study, and after correcting for BMI, age and sex, we found 85 ’omic measurements and clinical labs that correlated significantly with SSPG levels (Fig. 2b, Supplementary Table 8, *q* < 0.1); 75 were repeatedly observed using correlational analysis with insulin-resistant/insulin-sensitive classification (Extended Data Fig. 3c, Supplementary Table 9). As reported previously, triglycerides were positively associated with SSPG, whereas high-density lipoprotein (HDL) was inversely correlated with SSPG^15,16^. We also found that SSPG positively associated with increased inflammation and immune responses, as evident by neutrophil absolute count (*q* = 0.028) and white blood cell count (*q* = 0.066) from clinical laboratory tests. Although these complete blood count values were still in the normal range, these observations highlight the association between inflammation and insulin resistance^17,18^. Insulin resistance is also associated with altered lipid biology, and levels of several long-chain and polyunsaturated fatty acids correlated positively with SSPG. Notable metabolites whose levels correlated inversely with insulin-resistant/insulin-sensitive classification included indolelactic acid^19^ (*q* = 0.13) and hippuric acid (*q* = 0.043), which inversely correlate with metabolic syndrome and are strong markers of gut microbiome diversity^20^. Consistent with our metabolomics, the genus *Blautia*, which inversely correlates with hippuric acid, was positively correlated with SSPG (*q* = 0.045), in agreement with reports of a positive correlation between this genus and impaired glucose tolerance and diabetes^21^. On the other hand, the genera *Odoribacter* (*q* = 0.028), *Oscillibacter* (*q* = 0.011), and *Pseudoflavonifracter* (*q* = 0.0007) were negatively associated with SSPG, consistent with previous findings that *Odoribacter* is more abundant in healthy control individuals than in patients with non-alcoholic fatty liver disease^22^, in whom insulin resistance is common. Altogether, insulin resistance was associated with higher inflammation and altered lipid metabolism, which might cause insulin-resistant participants to have impaired responses to additional stresses, as reported previously^19^ and below.

## Pathways associated with respiratory viral infections

**Fig. 3:**
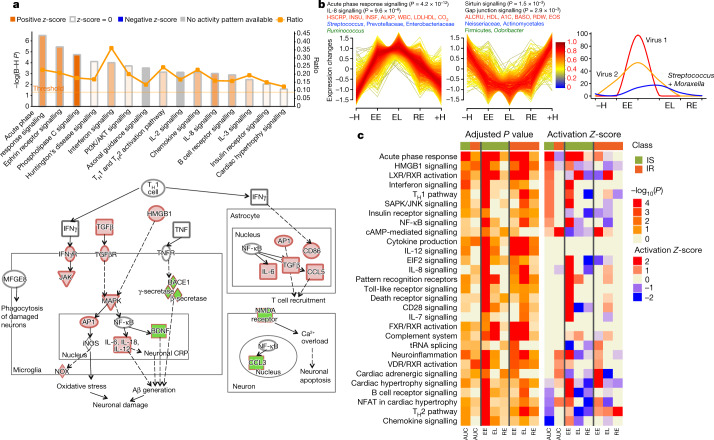
Dynamics of differential responses during RVI. **a**, Top, examples of the most significantly enriched integrated canonical pathways that showed differential changes in response to RVI according to the AUC test (two-sided). All *P* values were corrected using the Benjamini–Hochberg (B–H) method for multiple hypothesis correction. The Ingenuity Pathway Analysis (IPA) *Z*-score measures the match between expected relationship direction and observed gene expression. The ratio presents the percentage of molecules enriched from the total number of molecules within a pathway. Bottom, a simplified example of enriched neuroinflammation signalling showing molecules that were significantly upregulated (red) or downregulated (green) in response to RVI. The full pathway is shown in Extended Data Fig. 4d. **b**, Two time-series clusters (left and middle) that show up- and downregulation of significant ’omics molecules during RVI (AUC test, two-sided) with their top enriched pathways (black), clinical laboratory tests (red), gut microbial communities (green), and nasal microbial communities (blue). Trend lines are colour-encoded with red shades denoting high membership values of genes belonging to the time-series cluster. Right, a general time-series pattern was observed regarding viral load in relation to both nasal and gut microbial changes. The *y* axis denotes the percentage of maximal abundance. ALKP, alkaline phosphatase; INSU, insulin; INSF, insulin, fasting; LDLHDL, low-density to high-density lipoprotein ratio; ALCRU, aluminum/creatinine ratio random, urine; BASO, basophil in percentage; RDW, red blood cell distribution width; EOS, eosinophil in percentage; CO_2_, carbon dioxide. Virus 1 and Virus 2 denote generically for different viral species that changed in individuals. **c**, Heatmap showing different responses to RVI in the insulin-sensitive (77 RVI and 62 healthy categorized time points) and insulin-resistant groups (33 RVI and 17 healthy categorized time points) at the integrated pathway level based on the AUC test (two-sided) over the entire course of RVI and paired *t*-test (two-sided) for stage-wise comparison. Left, B–H corrected pathway *P* value enrichment heatmap. Right, activation *Z*-score heatmap. Heatmap bottom annotation: AUC, EE, EL and RE represent analysis over the entire course of RVI (AUC), at EE, EL and RE stages as compared to healthy baseline.

**Extended Data Fig. 4:**
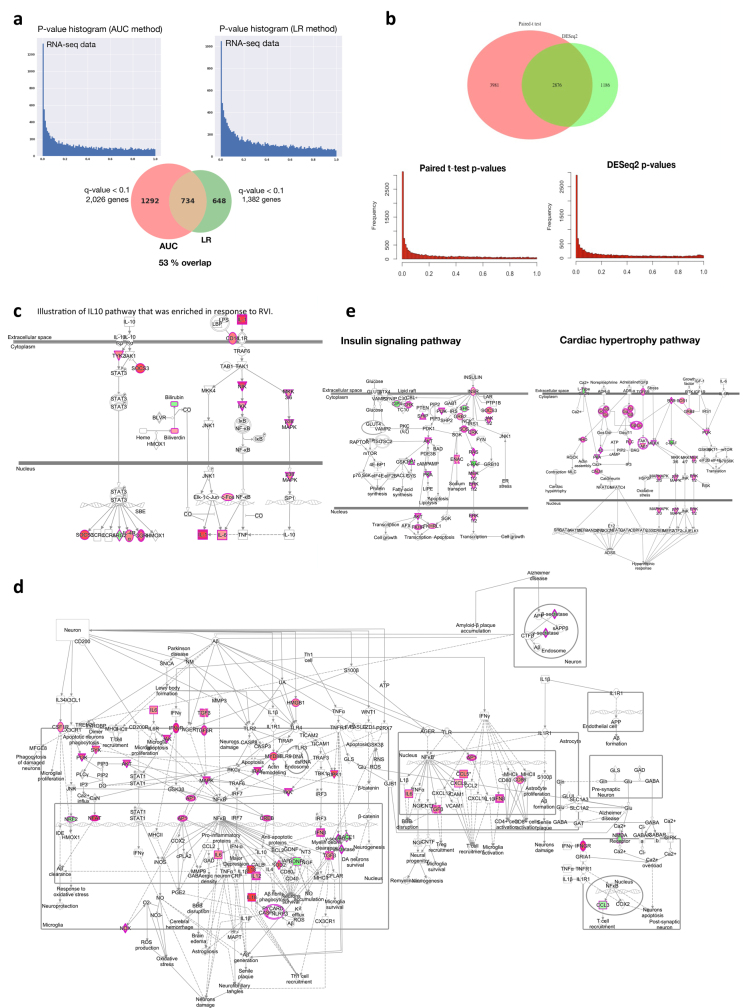
Differential responses to RVIs. **a**, Comparison of AUC test (two-sided) performance with LR analysis with the time covariate. Left, distribution plot of *P* values resulting from the AUC test. Right, distribution plot of *P* values resulting from the LR analysis. The pie chart shows the number of differentially expressed genes (*q* < 0.1) for both methods and the overlapping number. **b**, Top, comparison of results from paired *t*-test (two-sided) with DESeq2 method (Wald test) for stage pairwise analyses (for example, EE versus personal healthy baseline). Seventy-one per cent of transcripts identified by DESeq2 method overlap with paired *t*-test results. Bottom, *P* value distribution for paired *t*-test (left) and DESeq2 (right). Both methods show a homogenous distribution of *P* values with enrichment for significant ones. Supplementary Table 39 lists molecules found significant by paired *t*-test or DESeq2 method. **c**, Illustration of IL10 pathway enriched in response to RVI. Multi-omic molecules that changed significantly during the course of RVI by AUC test are highlighted in red (upregulated) or green (downregulated). **d**, An example of enriched neuroinflammation signalling showing molecules significantly upregulated (red) or downregulated (green) in response to RVI. Simplified pathway graph is shown in Fig. 3a (bottom). **e**, Illustration of insulin signalling (left) and cardiac hypertrophy (right) pathways, which were enriched in response to RVI. Molecules that changed significantly during the course of RVI by AUC test are highlighted in dark pink (upregulated) or green (downregulated).

To better understand the changes that occur during RVI, we grouped the time points into five categories: healthy time points before RVI (–H, less than 186 days before the first RVI visit), early events (EE, days 1–6 after infection), late events (EL, days 7–14 after infection), recovery (RE, days 15–40 after infection) and healthy time points after RVI (+H, less than 186 days after the last RVI visit; Extended Data Fig. 1c). To identify molecules that deviated significantly from healthy baselines (–H and +H) during RVI, we used area under the curve (AUC) statistics to test for differential expression over time and paired *t*-tests for category-specific changes (for example, EE versus baseline; see Methods, Extended Data Fig. 4a, b). We identified 2,026 transcripts, 11 cytokines, 145 metabolites, 29 proteins, 11 gut microbial taxa, 30 nasal microbial taxa and 25 clinical laboratory tests that differed significantly from personal baselines based on the AUC test (*q* < 0.1; Supplementary Table 10). Integrated canonical pathway analyses, based on transcripts, proteins, cytokines and metabolites, identified pathways associated with defence responses, such as interleukin signalling pathways, mTOR signalling^23^, and B cell and T cell receptor signalling^24^, among others^25^ (Fig. 3a, Supplementary Table 11, Extended Data Fig. 4c). We also identified the eukaryotic initiation factor 2 (eIF2) signalling pathway as induced early during RVI (EE stage); eIF2 gets activated by RVI and regulates pro-inflammatory cytokine expression^26^. Our analysis revealed molecular pathways that have, to our knowledge, not previously been shown to be dysregulated upon RVI (Fig. 3a). These include: 1) neurological pathways (for example, Huntington’s disease, *q* = 7.7 × 10^–5^; axonal guidance, *q* = 3.0 × 10^–4^; and neuroinflammatory signalling, *q* = 5.0 × 10^–2^; Fig. 3a, Extended Data Fig. 4d); 2) metabolic pathways (for example, insulin receptor signalling, *q* = 9.1 × 10^–3^; leptin signalling in obesity, *q* = 2.9 × 10^–2^); and 3) cardiac hypertrophy (*q* = 2.5 × 10^–2^; Extended Data Fig. 4e, Supplementary Table 11). Thus, a variety of biological pathways are altered during viral infection.

**Extended Data Fig. 5:**
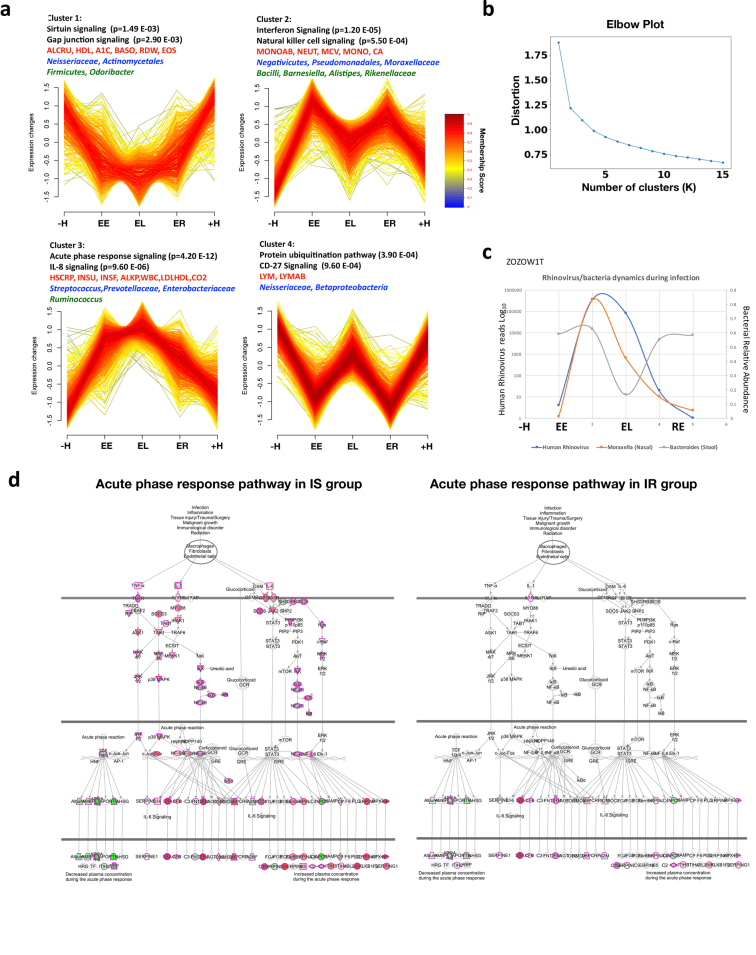
Trend analysis of differential changes in response to RVI. *n* = 156 RVI categorized time points and *n* = 89 healthy time points based on AUC test, two-sided. **a**, Four time-course patterns clustered by differential ’omic molecules that changed in response to RVI. **b**, The elbow method showed the optimal number of clusters (*K* = 4). **c**, Example of co-occurring changes in nasal and stool bacteria during upper respiratory rhinovirus infection. In subject ZOZOW1T, a high load of human Rhinovirus was found in RVI EE stage, accompanied by an increase in *Moraxella* (80%) in nasal samples. The abundances of Rhinovirus and *Moraxella* in nasal samples decreased in RVI EL and continued to decrease during the RE stage. Rhinovirus could not be detected at the next healthy time point and *Moraxella* showed a relatively low abundance (5%). *Bacteroides*, the dominant genus in the stool samples, decreased by 40% from EE to EL and returned to the level of the previous healthy time point at the RE stage. Rhinovirus was identified by nasal transcriptome and quantified by mapping the transcriptome reads to the virome references database. *Moraxella* and *Bacteroides* in nasal and stool samples were identified and quantified by 16S rRNA gene sequencing. **d**, Acute phase response pathway representation upon RVI in the insulin-sensitive group (left; *Z*-score 3.9 at EE stage) and the insulin-resistant group (right; *Z*-score 1.2 at EE stage). Upregulated molecules are highlighted in red, downregulated ones in green.

We further identified four major temporal clusters of molecules that were upregulated or downregulated during the course of RVI, as well as their top corresponding significant canonical pathways, microbial communities, and clinical laboratory data (Fig. 3b, Supplementary Table 12, Extended Data Fig. 5a, b, and Discussion). As expected, the level of HSCRP and white blood cell count increased and HDL^27^ decreased upon RVI. We also observed many changes in the gut and nasal microbiome, including order Firmicutes and genus *Odoribacter* in the gut, which have been reported to decrease in patients with inflammation and RVI^28,29^. There was also an increase in *Ruminococcus*, *Barnesiella*, *Alistipes*, Rikenellaceae and *Bacilli* in the gut. Nasal microbiota also changed during RVI. We observed a general trend of milder or delayed nasal bacterial changes following the viral load during RVI (Fig. 3b), with the nasal microbial changes being the opposite of those seen in the gut in one participant, for example (Extended Data Fig. 5c), which suggested that viral and bacterial changes in the nares and gut might be coordinated. An in-depth analysis of this phenomenon will be presented elsewhere (submitted).

We next compared the responses to RVI between insulin-resistant and insulin-sensitive participants. Insulin-resistant participants showed fewer changes after RVI than did insulin-sensitive participants (649 versus 873 significantly different molecules based on AUC test, *q* < 0.05, Supplementary Tables 13, 14; Supplementary Tables 17–19 for stage-wise tests), and the affected pathways were different between the two groups (Fig. 3c, Supplementary Tables 15, 16). Notably, most immune-related pathways were upregulated at the EE stage in insulin-sensitive participants, whereas almost no immune responses were evident until the EL stage in the insulin-resistant group. One key difference is that the acute phase response, which is a rapid inflammatory response that protects against infection using non-specific defence mechanisms, was activated and sustained through the EL stage in insulin-sensitive individuals but not in insulin-resistant individuals (Extended Data Fig. 5d). Furthermore, in agreement with the impaired host response in the insulin-resistant group, we observed fewer changes in nasal microbiota taxa and predicted genes in the insulin-resistant group during RVI. Notably, the nasal genus *Streptococcus* became more abundant during the course of RVI only in insulin-sensitive participants. Both the richness and the diversity of nasal microorganisms decreased significantly (*q* < 0.05) during RVI in insulin-sensitive but not insulin-resistant participants. It is possible that insulin-resistant participants have elevated baseline inflammation and an impaired immune response, which might hinder the coordination between the virus and bacteria during infection^30^. Moreover, changes in gut microorganisms differed between the insulin-resistant and insulin-sensitive groups (Supplementary Tables 13, 14). Overall, our results highlight the potential contribution of RVIs to the increased risk of developing metabolic disorders such as T2D in insulin-resistant individuals, and an impaired immune response to RVIs in these individuals.

## Distinct responses to immunization

**Fig. 4:**
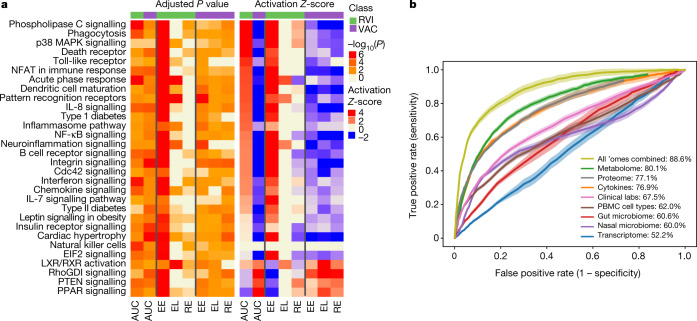
Temporal multi-omics responses to immunization. **a**, Heatmap showing different responses during RVI (*n* = 156 categorized time points) and immunization (*n* = 117 categorized time points, AUC test, two-sided, over the entire course of events and paired *t*-test, two-sided, for stage-wise comparison), same positions/labels as in Fig. 3. **b**, The ROC curve for classifiers designed to discriminate RVI time points from healthy baselines at different discrimination thresholds in testing data based on an LR model. 95% confidence intervals are shown as coloured ribbons. AUC scores of ROC curves are listed on the right for classification accuracy.

**Extended Data Fig. 6:**
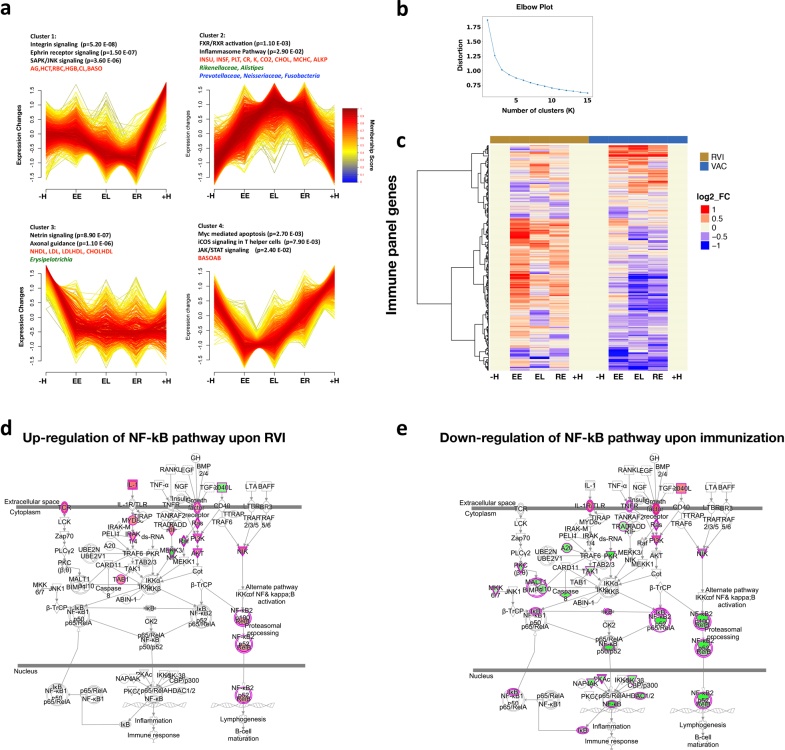
Trend analysis of differential changes in response to RVI and immunization. Data include 117 immunization (VAC) and 59 healthy categorized time points based on AUC test, two-sided. **a**, Four time-course patterns clustered by differential ’omic molecules that changed in response to immunization. **b**, The elbow method shows the optimal number of clusters (*K* = 4). **c**, Heatmap of immune molecules that changed significantly in response to RVI and immunization. The heatmap uses log_2_(baseline normalized read counts) to measure changes in expression in every category (EE, EL, RE) compared to the personal baseline (–H and +H). Red, upregulation; blue, downregulation. **d**, NF-κB pathway upregulation after RVI (*Z*-score = +1.96). **e**, NF-κB pathway downregulation upon immunization (*Z*-score = –4.9). Red, upregulated; green, downregulated.

**Extended Data Fig. 7:**
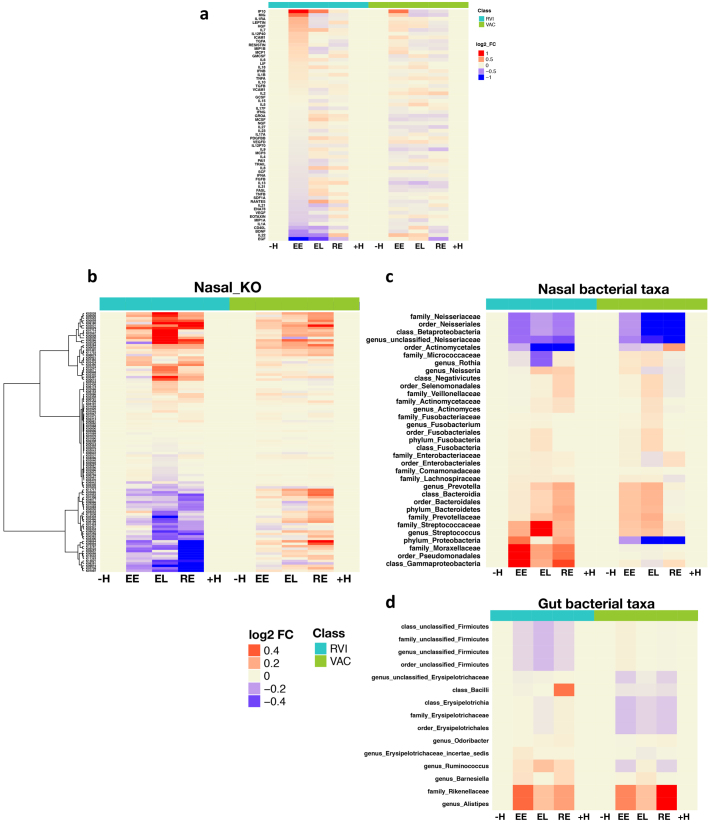
Trend analysis of differential changes in response to respiratory viral infections (RVI, *n* = 156) and immunization (VAC, *n* = 117). **a**, Heatmap of cytokine expression over the course of RVI and VAC. The heatmap uses log_2_(baseline normalized read counts) to measure changes in expression in every category (EE, EL, RE) compared to personal baseline (–H and +H). Red, upregulation; blue, downregulation. **b**–**d**, Heatmaps of nasal KO genes (**b**), nasal microbial taxa (**c**) and gut microbial taxa (**d**) over the course of RVI and VAC. The heatmaps use the log_2_(baseline normalized read counts) to measure changes in values in every category (EE, EL, RE) compared to the personal baseline (–H and +H).

Among responses to influenza immunization, we identified 3,019 transcripts, 9 metabolites, 6 proteins, 6 gut microbial taxa, 15 nasal microbial taxa and 23 clinical laboratory tests that differed significantly from personal baselines (*q* < 0.1; Supplementary Tables 20, 21; Supplementary Tables 23–25 for stage-wise tests). Temporal patterns in differentially expressed molecules over time are shown in Extended Data Fig. 6a, b and Supplementary Table 22. Although there were similarities in pathway enrichment between immunization and RVI (Supplementary Tables 11, 21), there were many striking differences (Fig. 4a). For example, integrated canonical pathway analysis identified changes in immune pathways, such as B cell receptor signalling and NF-κB signalling, that differed between RVI and immunization responses (Supplementary Tables 21, 26, Extended Data Fig. 6d, e). Moreover, regulators in most of these pathways, such as IFNγ, IL-2, IL-3, IL-6 and BDNF, behaved differently (as indicated by *Z*-score) between RVI and immunization (*q* < 0.05; Extended Data Fig. 6c). Our analyses also showed that upon immunization, the type 1 diabetes (T1D), type 2 diabetes (T2D) and insulin receptor signalling (*q* < 0.05) pathways were predicted to be downregulated (Fig. 4a, Supplementary Table 21). These observations are in contrast to the host response to RVI, and suggest that immunization might protect against T1D and T2D risk^31,32^. In addition, we identified 11 and 6 gut microbial taxa that were dysregulated during RVI and immunization, respectively (Supplementary Tables 10, 20, Extended Data Fig. 7). Among immunization-induced changes, the family Erysipelotrichaceae, which is associated with obesity^33^ and high fat intake^34^, was decreased, underscoring our observation that immunization inversely correlates with T1D and T2D signalling, which have overlapping biology with those metabolic disorders. These data reveal extensive differences in molecular pathway changes upon RVI and immunization, and identify dysregulated pathways that include immune responses, metabolic and neurological pathways, as well as gut and nasal microorganisms.

## Signatures for classification of stress events

**Extended Data Fig. 8:**
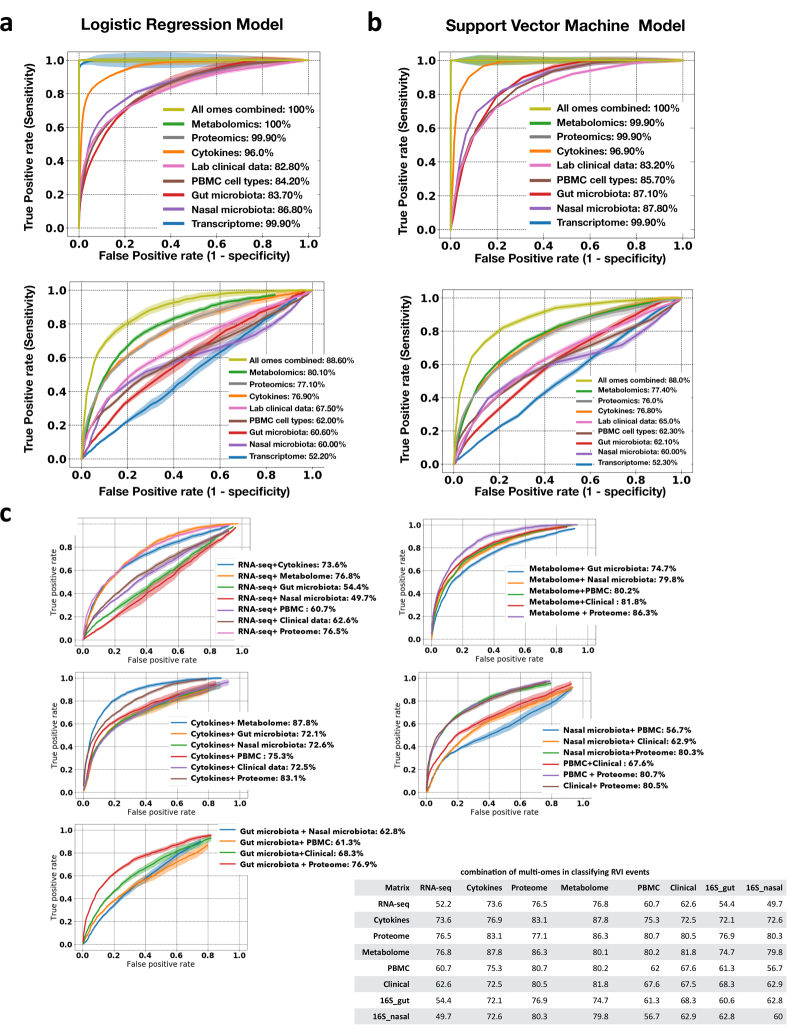
Receiver operating characteristic (ROC) curves for classifiers designed to separate RVI time points from healthy baselines at different discrimination thresholds. **a**, **b**, Performance in the training (top) and test sets (bottom) for LR (**a**) and SVM (**b**) models. Plots show the true positive rate (*y* axis) versus the false positive rate (*x* axis) for each ’ome and all ’omes combined. AUC scores for ROC curves are listed on the right for classification accuracy. For binary classification between healthy and RVI data, combined multi-omes had the highest prediction performance, followed by the metabolome, compared to others in the test cohort. **c**, ROC curves and AUC scores based on all-pairs testing using the LR model for classifying RVI events. The plot shows the true positive rate (*y* axis) versus the false positive rate (*x *axis) for each pairwise combination. Bottom right, summary of all pairwise combinations of multi-omes and their respective AUC scores in percentages.

**Extended Data Fig. 9:**
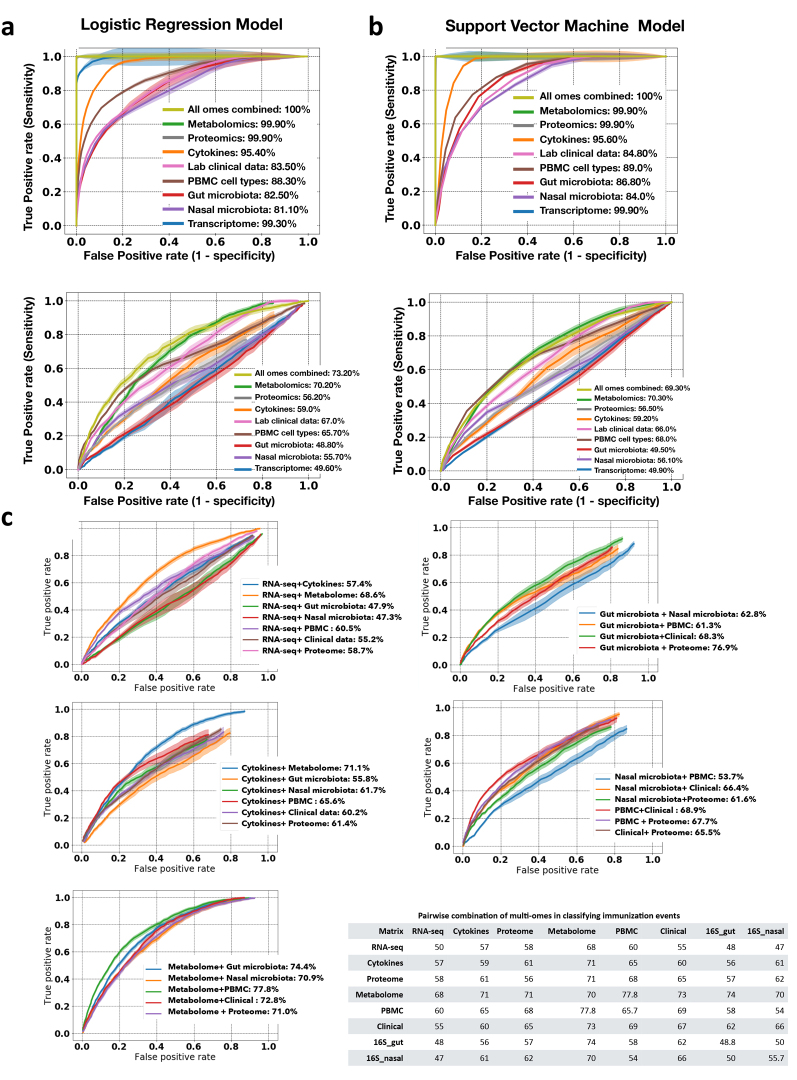
ROC curves for classifiers designed to separate immunization time points from healthy baselines at different discrimination thresholds. **a**, **b**, Performance in the training (top) and test sets (bottom) for LR (**a**) and SVM (**b**) models. Plots show the true positive rate (*y* axis) versus the false positive rate (*x* axis) for each ’ome and all ’omes combined. AUC scores of ROC curves are listed on the right for classification accuracy. For binary classification between healthy and immunization, combined multi-omes had the highest prediction performance, followed by metabolomes, compared to others in the test cohort. **c**, ROC curves and AUC scores based on all-pairs testing using the LR model for classifying immunization events. The plot shows the true positive rate (*y* axis) versus the false positive rate (*x* axis) for each pairwise combination. Bottom right, summary of all pairwise combinations of multi-omes and their respective AUC scores in percentages.

We further investigated the power of multi-omic data to classify stress: healthy baselines versus RVI (Fig. 4b, Extended Data Fig. 8), and healthy baselines versus immunization (Extended Data Fig. 9). The multi-omes, metabolome, proteome and cytokines had the highest classification performance for discriminating RVI from healthy time points (88.6%, 80.1%, 77.1% and 76.9% classification accuracy, respectively, in logistic regression (LR) models). The top most predictive ’omics features selected by both LR and support vector machine (SVM) models included IP10 (a pro-inflammatory cytokine), followed by serum amyloid A1, immature dendritic cells (a PBMC cell type), C_12_H_19_NO_2_ (metabolite), serum amyloid A2, EFEMP1, LYM (lymphocyte percentage), *GZMB* (transcript), urocanic acid, betaine, and *CD180* (transcript) (Supplementary Table 28). Using LR and SVM models to distinguish immunization from healthy time points (Extended Data Fig. 9, Supplementary Table 29), we revealed that multi-omes and metabolome markers had the highest accuracy (73.2% and 70.2%, respectively, in LR models). In addition, we defined the performances of pairwise combinations of multi-omes in classifying stress events from healthy baseline. The overall better performances of multi-omes, even with a smaller sample size, in classifying stress events from healthy baselines as compared to a single ’ome demonstrates the complementarity of different ’omes in describing the molecular processes that occur upon shifts in host physiological state.

## Molecular correlations across multi-omes

**Fig. 5:**
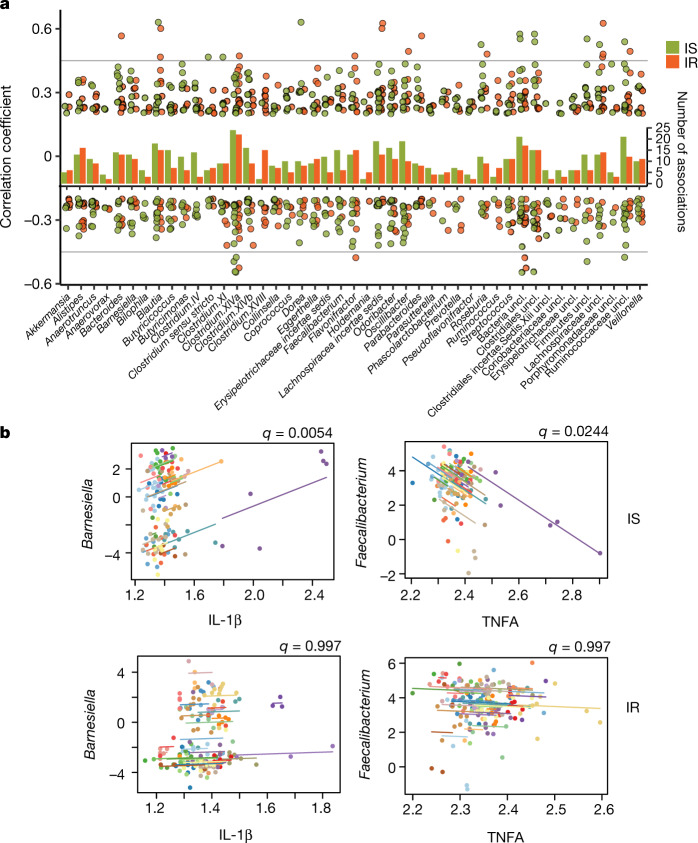
Correlational networks capture multi-omics association structures that differ between insulin-resistant and insulin-sensitive groups. **a**, Gut microbial associations are different in insulin-sensitive from insulin-resistant participants (insulin-sensitive *n* = 190 healthy visits, insulin-resistant *n* = 184 healthy visits, as not all visits had stool sampling). For each gut microorganism genus, significant associations (by SparCC) to other genera are displayed with their correlation coefficients as dots and the total number of significant associations (*q* < 0.05) as the bar in the middle. b, Examples of microbial-cytokines correlations (by CLR+rmcorr) that are significant in insulin-sensitive but not insulin-resistant participants. Longitudinal measurements and the correlation trend line are coloured per individual and *q* values are indicated at the top right of each comparison.

**Extended Data Fig. 10:**
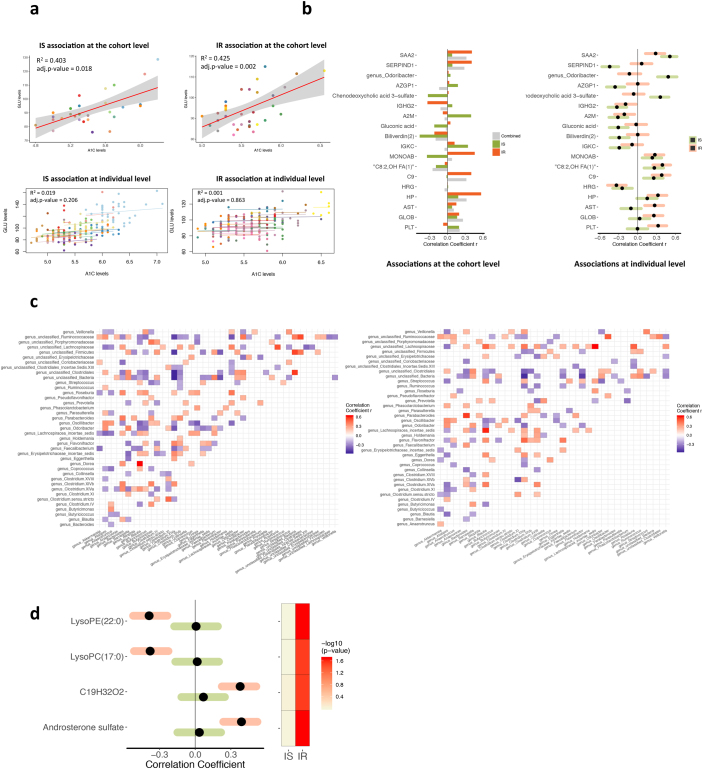
Associations among multi-omic molecules. Insulin-resistant *n* = 215, insulin-sensitive *n* = 238, all *n* = 624 including baselines from individuals with unknown insulin sensitivity, Rmcorr correlation. **a**, Comparison of associations between A1C and fasting glucose level (GLU) for between-individual associations (at the cohort level, top) and within-individual associations (at individual level, bottom) in insulin-resistant and insulin-sensitive groups. Correlation coefficients and adjusted *P* values (B–H corrected) are shown in each comparison. For comparison, data from individuals are coloured differently, with a single colour for all time points from the same individual. At the cohort level, the median fasting plasma glucose per participant correlated positively with the median haemoglobin A1C level for both insulin-sensitive and insulin-resistant groups. However, at the individual level, there was no significant correlation. This is expected, as fasting glucose is very variable and can be affected by diet or lifestyle within hours, whereas A1C approximates average glucose levels over about 3 months. **b**, Associations with HSCRP are shown either at the cohort level (left, between-individual correlation) or at individual level (right, within-individual correlation) for comparison between insulin-resistant and insulin-sensitive individuals. Only significant associations (*q* < 0.05) in insulin-resistant or insulin-sensitive subjects at individual level are used as examples. **c**, Significant (by SparCC) association patterns between gut microorganisms at the genus level in insulin-sensitive (left) and insulin-resistant individuals (right, insulin-sensitive *n* = 190, insulin-resistant *n* = 184, as not all visits included stool sampling). Red, positive correlation; blue, inverse correlation. **d**, An example of gut microorganisms: genus *Butyricimonas* and its significant associations with host metabolites (by CLR+rmcorr) that are specific to insulin-sensitive or insulin-resistant groups. Black dots denote the correlation coefficients of the genus related to corresponding metabolites on the left, with their 95% confidence intervals shown as either green ribbons for insulin-sensitive-specific or red ribbons for insulin-resistant-specific associations. The *P* values of those associations are shown on the right.

With thousands of multi-omic molecules measured simultaneously both in the human host and microorganisms, our study provides an opportunity to identify associations between molecules and pathways, and to compare them among insulin-sensitive and insulin-resistant participants. We interpreted associations at two levels for healthy visits: the personal level (within-individual by LME models; Fig. 5, Supplementary Table 30) and at the cohort level (cross-sectionally between individuals after correcting for BMI, age and sex; Supplementary Table 31), both of which provide different mechanistic perspectives (Extended Data Fig. 10a, b).

For intra-individual correlation analyses, we focused on associations of intra-omes and between gut microorganisms and other host omes for differences between insulin-sensitive and insulin-resistant groups. We found no significant differences in the average number of associations (that is, edges) per analyte between insulin-sensitive and insulin-resistant participants. However, insulin-sensitive participants had different analyte co-associations (significant, *q* < 0.05, and unique to the insulin-sensitive group) compared to those in insulin-resistant participants (Supplementary Table 30), including intra-associations among gut microorganisms (Supplementary Table 32). Insulin-sensitive and insulin-resistant unique and statistically significant associations for each gut microorganism are shown in Fig. 5a (pairwise association patterns in Extended Data Fig. 10c). Notably, the genus *Holdemania*, which might facilitate cancer immunotherapies^35^, was significantly associated with two other genera (*Clostridium.XlVb* and *Phascolarctobacterium*, *q* < 0.05) in insulin-sensitive participants, but significantly correlated with five other genera in insulin-resistant participants (including *Clostridium.XlVa*, *Clostridium.XVII* and *Collinsella*, *q* < 0.05; Supplementary Table 32). Such insulin-resistant-specific and insulin-sensitive-specific associations indicate that there are different patterns of gut microbial interactions in the two groups.

We also examined correlations between gut microorganisms and host metabolites. The total number of associations differed between insulin-sensitive and insulin-resistant participants, confirming previous findings^18^ (Supplementary Table 33). In the correlations between gut microorganisms and host circulating cytokines, we observed five significant insulin-sensitive-specific associations (*q* < 0.05) but no insulin-resistant-specific associations; for example, the genus *Barnesiella* was positively associated with IL-1β (*q* = 0.0054) and the genus *Faecalibacterium* was inversely associated with TNFA (*q* = 0.0244) only in insulin-sensitive subjects (Fig. 5b). Because *Barnesiella* is important in host IL-1 activity^36^ and IFNγ signalling^37,38^ and *Faecalibacterium* is linked to TNF^39^, which might synergize with IL-17 activity^37,40^, the lack of these associations in insulin-resistant participants suggests that insulin resistance may affect interactions between the gut microbiome and host cytokines. Furthermore, the genus *Butyricimonas* was negatively associated with four lipids only in insulin-resistant participants (*q* < 0.05, Extended Data Fig. 10d), highlighting this previously reported bacterial relationship with lipid metabolism^41^; our finding indicates that this relationship is also relevant to glucose dysregulation. Together, these observations indicate that gut microorganisms and host immunity and metabolism are coordinated differently in insulin-sensitive and insulin-resistant participants.

## Longitudinal tracking of diabetes in individuals

**Fig. 6:**
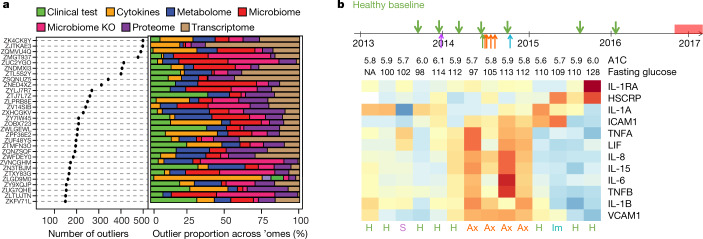
Personal features that precede the onset of T2D revealed by longitudinal tracking. **a**, Left, scatter plot showing the number of outliers among thousands of molecules for each participant; right, the percentage of outliers contributed by each ome. **b**, Top, collection timeline for participant ZNED4XZ in our study relative to onset of T2D, with green arrows pointing to eight healthy baselines (H), purple for one stress visit (S), red for four antibiotics visits (Ax) and blue for one immunization visit (Im). Bottom, heatmap showing levels of selected immune cytokines together with A1C (%) and fasting glucose (mg dl^−1^) across time. Red denotes increased expression; blue represents decreased expression, with shades corresponding to levels.

**Extended Data Fig. 11:**
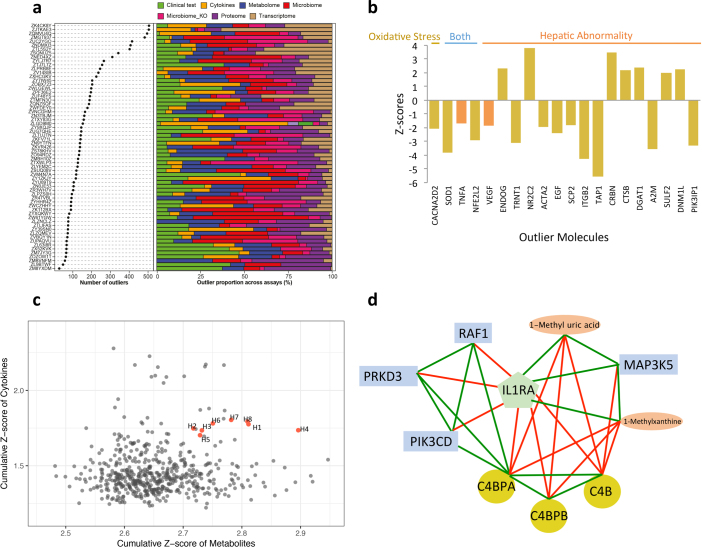
Personal features reveal molecular markers before the onset of diseases. **a**, Left, scatter plot showing the number of outliers among thousands of molecules for each participant; right: the percentage of outliers contributed by each ’ome. The top part of the plot is also shown in Fig. 6a. **b**) *Z*-scores of outlier molecules in participant ZJTKAE3. **c**, Metabolite–cytokine profiles of the cohort. All healthy baselines (H1–H8) of participant ZNED4XZ are highlighted in red. **d**, Correlational network among molecules that were highly associated with IL1RA and enriched in xenobiotic-induced immune response pathways. Metabolites, orange ovals; transcripts, blue rectangles; proteins, yellow squares. Positive associations, green lines; negative associations, red lines.

Analysis of the multi-omic profiles revealed a unique set of molecules for each individual that was different from the cohort mean (Fig. 6a, Supplementary Table 34). For example, one individual (participant number ZJTKAE3) had a high number of outlier transcripts, and pathway enrichment analysis suggested that these were enriched in toxicity pathways, especially in oxidative stress and hepatic abnormality (Extended Data Fig. 11a, b, Supplementary Table 35). It is possible that this individual started to develop hepatic or related abnormalities at the molecular level even while symptom-free. Indeed, this participant was diagnosed five months later with mild hepatic steatosis^12^.

Longitudinal analysis of another participant (ZNED4XZ) revealed a number of ‘healthy’ samples that contained outliers in both metabolite and cytokine profiles compared to the rest of cohort (Extended Data Fig. 11c, Supplementary Table 36). This participant was clinically diagnosed with T2D by an independent hospital 10 months after her last study visit, and our data suggest a molecular mechanism of her T2D onset. Among a curated list of immune molecules, interleukin-1 receptor antagonist (IL-1RA) and HSCRP were highly elevated during her last three healthy visits before T2D diagnosis (Fig. 6b). Notably, 262 additional molecules followed the same trend as IL-1RA (and 75 for HSCRP) along the participant’s path to T2D (Supplementary Tables 37, 38). Among molecules that were highly correlated with IL-1RA, we found a signature of xenobiotics, including methyluric acid and methylxanthine, which are gut microorganism-associated metabolites related to impaired glucose tolerance^42^ (Extended Data Fig. 11d). Those xenobiotics were also tightly associated with expression of host factors involved in the complement system (C4B, C4BPB, C4BPA), acute immune response signalling (*PIK3CD*, *MAP3K5*), and the LPS-stimulated MAPK pathway (*RAF1*, *PRKD3*, *MAP3K5* and *PIK3CD*; Extended Data Fig. 11d), all of which are associated with the development of diabetes^43,44^. Moreover, we found a decrease in gut microbial diversity and weight increase before T2D diagnosis^12^. Thus, by examining multi-omic measurements during the progression to T2D, we identified hundreds of molecules in which changes preceded the diagnosis of the disease and that are potentially associated with mechanisms underlying the development of T2D in this individual. Additional examples of health-related discoveries can be found in a companion paper^12^.

## Discussion

To better understand how healthy individuals and those at risk of T2D change over time and in response to perturbations, we profiled both healthy quarterly baselines and other stress visits (for RVI, immunization, antibiotics and others) for 106 participants over nearly four years. This work provides a unique data set that can be used to understand variation both across the cohort and at an individual level among healthy baselines and in response to different stresses. For each multi-omic measurement, we identified the source of healthy variation and studied the variability within and between participants (Supplementary Tables 4, 5). Although molecules measured in clinical laboratory tests and other omes varied between individuals, overall they were relatively stable within each individual. Notably, even though the levels of these highly personal molecules may deviate during occurrence of a disease, they are still often within the population ‘healthy’ range and thus are not diagnosed in an individual using population measurements. The strong interpersonal difference among individuals is likely to be the reason why large numbers are required for detecting differences between cases and controls in disease studies. The distinct personal baselines identified in this study may be due to the genetic, epigenetic, and/or environmental (for example, lifestyle, diet preference, exercise level) differences between participants.

Beyond healthy baselines, we examined dynamic changes in host molecular pathways and microbiota communities during RVI and immunization. During RVI, our integrated multi-omics approach revealed activation of defence mechanisms that are associated with innate immunity and adaptive responses. Notably, we identified three non-respiratory effects that have not been well explored in connection to RVI. These include activation of neurological pathways (associated with neuroinflammation, Huntington’s disease, and axonal guidance), cardiac hypertrophy, and metabolic pathways (for example, insulin receptor signalling and leptin signalling in obesity). Multiple epidemiological and clinical reports have described an association of RVI outbreaks with neurological diseases specifically in children, leading to an emerging hypothesis that inflammation from respiratory viral infections can promote chronic neuronal dysfunction^45^. The relation between cardiac complications and RVI is complex. Association of cardiac complications with influenza infection in adults has been reported^46^. However, in most of these cases cardiac complication is relatively reversible, as we found in our study. The third extra-respiratory effect was on metabolic pathways such as insulin receptor signalling and T2D pathways. Although recurrent RVI has been associated with type 1 diabetes, this has not been well investigated for T2D except one case^7^; thus, our work extends this observation. As these signatures were identified by analysing blood components, they are likely to represent systemic effects.

Notably, the insulin-resistant and insulin-sensitive groups differed at baseline and during stress responses. Insulin-resistant participants had higher triglycerides as well as an increased level of immune molecules, consistent with previous findings^14,18^. Insulin-resistant participants had an impaired and delayed response to RVI, and this impairment can lead to chronic inflammation resulting in T2D^47^. In agreement with the impaired host response in the insulin-resistant group, nasal and gut microbiota in the insulin-resistant group had a milder response to RVI than in the insulin-sensitive group. Furthermore, host–microbiome associations are affected by insulin-resistance status.

T2D is an inflammatory disease, and several inflammation molecules are important indicators of T2D development, including IL-1RA^48^, HSCRP, IL-6^49^, IL-1β and TNF^50^. In the case of participant ZNED4XZ, only IL-1RA and HSCRP increased before the onset of T2D, but not the other markers, including IL-6 and IL-1β. Because IL-6 and IL-1β are mostly produced by leukocytes in the liver and adipose tissue, it is likely that the development of T2D in this participant was not caused by liver or adipose inflammation at those time points. Longitudinal tracking and profiling of participant ZNED4XZ demonstrated the power to decipher disease aetiology at an individual level, which might be more specific than the general understanding gained from population measurements. Additional work is required to assess how frequently the mechanism we propose in this participant also applies to other cases of T2D.

Overall, these results point to a complex and dynamic ’omic landscape in both host and microorganisms during health and perturbations such as RVI and immunization. They further reveal how insulin-resistant and insulin-sensitive participants differ both at baseline and in their responses to RVI. These deep multi-omics measurements enable us to investigate the early molecular signs of disease development at an individual level. Future efforts will help to provide additional mechanistic understanding of how the multi-omic factors affect health and are altered early in disease development, both at the cohort and individual levels.

## Methods

### Participant recruitment and IRB consent

Participants provided informed written consent for the study under research study protocol 23602 approved by the Stanford University Institutional Review Board. All participants were studied after an overnight fast at the Stanford Clinical and Translational Research Unit (CTRU). This study complies with all relevant ethical regulations and informed consent was obtained from all participants.

Participants were recruited through placement of advertisements in local newspapers and radio stations seeking ‘prediabetic volunteers’ at risk of T2D for a longitudinal multi-omic study. Screening in the CTRU entailed collection of clinical history, physical examination, anthropometric measurements, and fasting blood tests for exclusions including presence of anaemia defined as haematocrit <30, renal disease defined as creatinine >1.5, history of any cardiovascular, malignancy, chronic inflammatory or psychiatric disease, and history of any bariatric surgery or liposuction. Study survey data were collected and managed using REDCap electronic data capture tools hosted at Stanford University.

Each eligible, consented subject underwent one-time quantification of insulin-mediated glucose uptake via the modified insulin suppression test as previously described and validated^10,51^. In brief, following an overnight fast, subjects were infused for 180 min with octreotide (0.27 µg/m^2^ min), insulin (25 mU/m^2^ min), and glucose (240 mg/m^2^ min). Blood was drawn at 10-min intervals from 150 to 180 min of the infusion to measure plasma glucose (oximetric method) and insulin (radioimmunoassay) concentrations: the mean of these four values comprised the SSPG and insulin concentrations for each individual. At steady state, insulin concentrations (65 μU/ml) were similar in all subjects and the SSPG provides a direct measure of the relative ability of insulin to dispose of a glucose load: the higher the SSPG concentration, the more insulin-resistant the individual. While the SSPG is distributed continuously, for the purpose of this study, we defined insulin-sensitive as SSPG < 150 mg/dl and insulin-resistant as SSPG ≥ 150 mg/dl, largely to provide separation between the two groups. In some cases SSPG tests were not performed, for reasons including: 1) participants withdrew from the study before their scheduled tests; 2) participants opted out of (that is, did not consent to) this test owing to their own schedule conflicts or other personal reasons; 3) participants were not eligible for the test as were already diagnosed as having diabetes (the IRB will not allow this).

### Blood sample preparation

Blood was drawn from overnight-fasted participants at the indicated time points at the Stanford CTRU. An aliquot of blood was incubated at room temperature to coagulate; clots were subsequently pelleted and the serum supernatant was pipetted off and immediately frozen at –80 °C. Blood from separate EDTA tubes was immediately layered onto Ficoll medium and spun via gradient centrifugation. The top layer of plasma was pipetted off, aliquoted and immediately frozen at –80 °C. The PBMC layer was removed and counted using a cell counter, and aliquots of PBMCs were further pelleted and flash-frozen with DMSO/FBS. For the subsequent multi-omic analyses, PBMCs were thawed on ice, and subsequently lysed and processed to DNA, RNA and protein fractions using Allprep Spin Columns (Qiagen) according to the manufacturer’s instructions and using the Qiashredder lysis option. Plasma analysis was performed on individual aliquots to prevent freeze–thaw cycles.

### Exome sequencing

In brief, DNA was isolated from blood using Gentra Puregene Kits (Qiagen) according to the manufacturer’s instructions. Exome sequencing was performed in a CLIA- and CAP-accredited facility using the ACE Clinical Exome Test (Personalis). Variant calling was performed using an in-house developed automated pipeline^52^.

### RNA sequencing

The transcriptome was evaluated using RNA-seq of bulk flash-frozen PBMCs. We used Qiagen All prep kit to extract total RNA from PBMCs according to the manufacturer’s protocol. The RNA libraries were constructed using the TruSeq Stranded total RNA LT/HT Sample Prep Kit (Illumina) with 500 ng total RNA, according to the manufacturer’s instructions. In brief, ribosomal RNA was depleted from total RNA using Ribo-Zero magnetic beads, then the ribosomal RNA-depleted RNA was purified and fragmented. A random primer tailed with an Illumina adaptor was used to perform reverse transcription to obtain a cDNA library. An adaptor sequence was added to the other end of the cDNA library with a Terminal-Tagging step. The cDNA library was amplified using the Illumina primers provided with this kit. Liquid handling was performed with an Agilent Bravo Automated Liquid Handling Platform. RNA-seq libraries were sequenced using the Illumina HiSeq 2000 (Illumina) instrument per the manufacturer’s instructions. Each library was quantified using an Agilent Bioanalyzer and Qubit Fluorometric quantification (ThermoFisher) using a dsDNA high-sensitivity kit. Quantified, barcoded libraries were normalized and mixed at equimolar concentrations into a multiplexed sequencing library. Sequencing of libraries was performed up to 2 × 101 cycles. We sequenced on average 5–6 libraries per lane of HiSeq2000. Image analysis and base calling were performed with the standard Illumina pipeline.

The TopHat package^53^ was used to align the reads to the hg19 reference genome and personal exome, followed by HTseq and DESEQ2 for transcript assembly and quantification of RNA expression^54,55^. Custom scripts in R and Python were used for downstream analyses. For data preprocessing, we first removed genes with average read counts over all samples smaller than 0.5. Then, samples with average read counts over all filtered genes smaller than 0.5 were filtered out.

The input file contained 883 samples as columns and 25,364 genes as rows. After filtering steps, we had 883 samples with expression data from 13,379 genes. For global variance and correlation analyses, in order to reduce the number of features, we removed genes with average read counts less than 1, resulting in 10,343 genes. We also used the xCell algorithm^56^ to deconvolute PBMC cell types based on RNA-seq data. For computation of abundance scores with xCell, all gene expression data were concatenated into a single file. Abundance scores were then computed from the expression data using xCell (xCellAnalysis function run with the ‘rnaseq =TRUE’ option and *N* = 64). The abundance scores of PBMC deconvoluted cell types were then used to classify stress events.

### Microbiome sampling

Sampling of stool, nasal, tongue and skin microbiomes was conducted according to the Human Microbiome Project – Core Microbiome Sampling Protocol A (https://www.hmpdacc.org/). Once samples were received in the laboratory, they were subsequently stored at –80 °C until further processing.

### Microbiomics

#### DNA extraction

DNA extraction was performed following Human Microbiome Project – Core Microbiome Sampling Protocol A (HMP Protocol # 07-001, v12.0). Metagenomic DNA was isolated in a clean hood using the MOBIO PowerSoil DNA Extraction kit, with added proteinase K, followed by lysozyme and staphylolysin treatment.

#### Targeted rRNA gene amplification and sequencing

For 16S (bacterial) rRNA gene amplification, hyper-variable regions V1–V3 of 16S were amplified from the metagenomic DNA using primers 27F and 534R (27F:5′-AGAGTTTGATCCTGGCTCAG-3′ and 534R: 5′- ATTACCGCGGCTGCTGG-3′). The oligonucleotides containing the 16S primer sequences also contained an adaptor sequence for the Illumina sequencing platform. A barcode sequence unique to each sample was embedded within each of the forward and reverse oligonucleotides used to create the amplicons (dual tags). The uniquely barcoded amplicons from multiple samples were pooled and sequenced on the Illumina MiSeq sequencing platform using a V3 2 × 300 sequencing protocol. The 16S rRNA gene is about 1.5 kb, and includes nine variable regions that provide much of the sequence distinction between different taxa. Variable regions 1–3 are generally sufficient to identify taxa down to the genus level, and sometimes to the species level. Illumina’s software handles the initial processing of all the raw sequencing data. One mismatch in primer and zero mismatch in barcodes were applied to assign read pairs to the appropriate sample within a pool of samples. Barcodes and primers were removed from the reads. Reads were further processed by removing sequences with low quality (average qual <35) and ambiguous bases (Ns). Chimeric amplicons were removed using UChime, and amplicon sequences were clustered and operational taxonomic units (OTU) picked by Usearch against GreenGenes database (May 2013 version). Final taxonomic assignment was performed using RDP-classifier. All details were executed using QIIME^57^ with custom scripts.

#### Metagenomic shotgun sequencing

DNA was also subject to whole genome metagenomic shotgun sequencing (mWGS) for a selection of nasal swabs during infection to identify bacteria and viruses to the species level (described in more detail in an accompanying paper^39^). The libraries were prepared according to a standard protocol from Illumina, and at least 1 Gb of 150 bp pair-end reads per sample was sequenced on the Illumina HiSeq or MiSeq instrument. Downstream processing of the mWGS reads included a) identification and masking of human reads (using NCBI’s BMTagger, ftp://ftp.ncbi.nlm.nih.gov/pub/agarwala/bmtagger); b) removal of duplicated reads that are artefacts of the sequencing process; c) trimming low-quality bases; and d) low-complexity screening (b–d were done through PRINSEQ). Reads trimmed to less than 60 bp were removed and the remaining high-quality reads were analysed downstream.

### Untargeted metabolomics by LC–MS

Plasma samples were prepared and analysed in a randomized order as previously described^58^. In brief, metabolites were extracted using 1:1:1 acetone:acetonitrile:methanol, evaporated to dryness under nitrogen and reconstituted in 1:1 methanol:water before analysis. Metabolic extracts were analysed four times using hydrophilic interaction (HILIC) and reverse-phase liquid chromatography (RPLC) separation in both positive and negative ionization modes. Data were acquired on a Thermo Q Exactive plus mass spectrometer for HILIC and a Thermo Q Exactive mass spectrometer for RPLC. Both instruments were equipped with a HESI-II probe and operated in full MS scan mode. MS/MS data were acquired on quality control samples (QC) consisting of an equimolar mixture of 150 randomized samples from the study. HILIC experiments were performed using a ZIC-HILIC column (2.1 × 100 mm, 3.5 μm, 200 Å; Merck Millipore) and mobile phase solvents consisting of 10 mM ammonium acetate in 50/50 acetonitrile/water (A) and 10 mM ammonium acetate in 95/5 acetonitrile/water (B). RPLC experiments were performed using a Zorbax SBaq column (2.1 × 50 mm, 1.7 μm, 100 Å; Agilent Technologies) and mobile phase solvents consisting of 0.06% acetic acid in water (A) and 0.06% acetic acid in methanol (B).

#### Metabolomics data processing

Metabolic extracts from 979 samples were prepared in a randomized order and data were acquired in three batches. LC–MS data were processed using Progenesis QI (Nonlinear Dynamics). Metabolic features from blanks and that did not show sufficient linearity upon dilution were discarded. Only metabolic features present in >33% of the samples were kept for further analysis and missing values were imputed using the *k*-nearest neighbours method. MS signal drift with time for non-targeted data cannot be easily corrected by using a small number of internal standards, as the drift is nonlinear and metabolite-dependent. To circumvent this issue, we applied LOESS (locally estimated scatterplot smoothing) normalization to our data. Each metabolic feature signal drift with time was independently corrected by fitting a LOESS curve to the MS signal measured in QCs. QCs were injected every 10 biological samples and consisted of an equimolar mixture of 150 random samples from the study. We showed that LOESS normalization was efficient to correct intra- and inter-batch metabolite-specific signal drifts as in Extended Data Fig. 1e. After further pre-processing and annotation of the metabolic features, a total of 722 metabolites were measured using our metabolite profiling platform, among which 431 were identified by matching retention time and fragmentation spectra to authentic standards or by comparing fragmentation spectra to public repositories.

### Proteomics (SWATH-mass spectroscopy)

Tryptic peptides of plasma samples were separated on a NanoLC 425 System (SCIEX). The flow was 5 μl/min with trap-elute setting using a 0.5 × 10 mm ChromXP (SCIEX). The LC gradient was set to a 43-min gradient from 4–32% B with 1 h total run. Mobile phase A was 100% water with 0.1% formic acid. Mobile phase B was 100% acetonitrile with 0.1% formic acid. We used an 8-μg load of undepleted plasma on a 15-cm ChromXP column. MS analysis was performed using SWATH acquisition on a TripleTOF 6600 system equipped with a DuoSpray source and 25-μm ID electrode (SCIEX). Variable Q1 window SWATH Acquisition methods (100 windows) were built in high-sensitivity MS/MS mode with Analyst TF Software 1.7.

#### Proteomics data processing

Peak groups from individual runs were statistically scored with pyProphet^59^ and all runs were aligned using TRIC^60^ to produce a final data matrix with 1% FDR at peptide level and 10% FDR at protein level. Protein abundances were computed as the sum of the three most abundant peptides (top3 method). A major maintenance of the mass spectrometer caused considerable batch effects across measured samples. To reduce batch effects, we performed subtraction of the principal components showing a major batch bias using Perseus^61^ 1.4.2.40.

### Luminex assays

Levels of circulating cytokines in the blood were measured using a 63-plex Luminex antibody-conjugated bead capture assay (Affymetrix) that has been extensively characterized and benchmarked by the Stanford Human Immune Monitoring Center (HIMC). Human 63-plexes were purchased from eBiosciences/Affymetrix and used according to the manufacturer’s recommendations with modifications as described below. In brief, beads were added to a 96-well plate and washed using a Biotek ELx405 washer. Samples were added to the plate containing the mixed antibody-linked beads and incubated at room temperature for 1 h followed by overnight incubation at 4 °C with shaking. Cold and room-temperature incubation steps were performed on an orbital shaker at 500–600 r.p.m. After the overnight incubation, plates were washed using a Biotek ELx405 washer and then biotinylated detection antibody added for 75 min at room temperature with shaking. The plate was washed as above and streptavidin-PE was added. After incubation for 30 min at room temperature, a wash was performed as above, and reading buffer was added to the wells. Plates were read using a Luminex 200 instrument with a lower bound of 50 beads per sample per cytokine. Custom assay control beads by Radix Biosolutions were added to all wells. Results from different batches were further corrected using controls and replicates shared between batches. The assay was performed by Stanford HIMC.

### Multivariate data analysis

Data matrices from all ’omics (clinical laboratory test, cytokines, transcriptome, MS-based proteome, metabolome, microbiome 16S data and WGS) were obtained and processed into a common format. Metabolites and protein MS intensities were log-transformed, while transcripts read counts were log_2_(*n* + 1) transformed. Only microbial taxa that were present (>0) or microbial genes with >0.1% abundance in more than a half of the entire collection (>400) were used further for downstream analysis. Microbial taxa and genes were arcsine transformed for downstream linear analyses^62,63^. Analyses for generating reported results are further specified below.

### Cohort interquartile range (IQR) and individual IQR

To evaluate the dispersion pattern of values per analyte across the whole cohort and within each subject, we used IQR to describe this variation. Analytes were first standardized with a standard deviation of 1 centred at 0 before applying IQR(Exp, na.rm = TRUE, type = 8) from the R stats package, where Exp was the linearly transformed and standardized values of each analyte. Values from all healthy visits of the entire cohort regardless of subjects were used to calculate the cohort IQR while only healthy visits of the corresponding subject were used for that individual’s IQR. The density plots of IQR distribution for each ’ome were visualized by geom_density() in R ggplot2 package.

### Healthy baseline variance decomposition

As each subject was sampled repeatedly at healthy visits, we used LME models to account for this dependence within subjects. We modelled random intercepts but a fixed slope, allowing different personal levels between subjects. We first linearly transformed each analyte (when applicable) and standardized the total variation to 1 before applying lmer() function from ‘lme4’ R package, with formula as: lmer(Exp ~ 1 + Days + A1C + SSPG + FPG + (1|SubjectID), data = data set, REML = FALSE), in which Exp was the linearly transformed and standardized values of each analyte. We thereby used intra-class correlation (ICC) as the proportion of total variation explained by subject structure in the cohort by **V**random subject/**V**total, in which **V** was variance from the corresponding component extracted by VarCorr() and **V**total was 1. Variations explained by fixed factors (Days, A1C, SSPG and FPG) were extracted by anova().

### Healthy MDS profiles influenced by personal factor

Either top ’omic molecules with the most individual contribution (highest in ICC) or molecules from each ’ome were used for further multidimensional scaling (MDS) analysis. The distance among healthy visits were calculated using Manhattan method in metaMDSdist() function from R vegan package and the MDS analysis using metaMDS() with *k* = 2. We calculated an individuality score (ind_score) by using the median value of healthy baselines in each individual and averaging pairwise distance among all individuals across molecules in question. Therefore, a higher ind_score and thus a larger average distance indicates a more distinct inter-personal pattern.

### Associations with time

We first used the first healthy visit as the reference baseline for each subject. The delta changes in the expression values in subsequent healthy visits were used to correlate with the delta change in days. Healthy visits from subjects with at least three healthy visits were used to assess the time associations. As each subject was sampled repeatedly at healthy visits, we used LME models to account for this dependence within subjects. We used rmcorr^64^, a method that is close to a null multilevel model of varying intercept and a common slope for each individual, and specifically tests for a common association between variables within each subject. Rmcorr calculates an effect size to appropriately represent the degree to which each subject's data are reflected by the common slope of the best-fit parallel lines. The rmcorr method takes meta-analytic approach and calculates rrm (error degrees of freedom), *P* value (determined by the *F*-ratio: *F*(Measure df (1), Error df)), and a 95% confidence interval of effect sizes (95% CI). When the relationship between variables varies widely across subjects, the rmcorr effect size will be near zero with confidence intervals also around zero. When there is no strong heterogeneity across subjects and parallel lines provide a good fit, the rmcorr effect size will be large, with tight confidence intervals. Additionally, to avoid potential bias introduced by subjects who had too few baseline profiles, analyses were also performed using a subset of 27 subjects who had more than 900 days profiled to compare. rrm correlation was further corrected for multiple hypothesis testing by FDR.

### Associations with SSPG or difference between insulin-resistant and insulin-sensitive in healthy baseline

We used between-individual correlation approach for this (see ‘Correlation network analysis’ section below). The median values of all healthy baselines per subject were used to associate their SSPG values or to compare insulin-resistant with insulin-sensitive results. Pearson correlation was used after linear transformation, normalization and correction for BMI, age and sex using the R ppcor package. For insulin-resistant/insulin-sensitive correlation, insulin-resistant/insulin-sensitive classification was first applied as dummy variables (insulin-sensitive as 0, insulin-resistant as 1) before further analyses. Additionally, with the method correcting BMI, age and sex, we tested the correlation with potential confounding factors, such as HDL, triglycerides, and triglycerides/HDL ratio, with SSPG^10,15^ as well as medication status on statin and any glucose control medication.

### ’Omics differential signatures during stress events

In order to identify temporal changes in ’omics molecules that deviated from the personal baseline over the course of stress events, we implemented the area under the curve (AUC) test. We defined longitudinal categories as follows: pre-healthy (–H) state (healthy baselines within 186 days before the event’s onset), event early (EE) state (visits on days 1–6 of the event), event late (EL) state (visits on days 7–14 since the event started), recovery (RE) state (visits within days 15–40 since the event started), and finally post-healthy (+H) state (visits within 186 days after the event; Fig. 3a).

The AUC test calculates the sum of means of each group (EE, EL, RE) after personal baseline correction, where the variation of the correction was also taken into account. As its name indicates, the AUC corresponds to area under the curve during the stress response over the five categorized time points (–H, EE, EL, RE, +H). This quantity can be interpreted as the total amount of change in the expression or abundance of molecules that deviate from the personal baseline over the entire course of RVI or immunization.

For each ’omics feature, the null hypothesis is that the mean expression level remains the same over the course of RVI or immunization. Under the null hypothesis, for each stress event we assume:

$${X}_{i,\alpha } \sim N\left({\mu }_{i},{\sigma }^{2}\right)$$

for individual *i* and category *α*∈{EE, EL, RE}. That is, the mean expression level depends on the individual but does not depend on the event type. For each sample with an event category *X*_*i,α*_, *α*{EE, EL, RE}, the personal baseline is corrected by subtracting the mean of the healthy time points next to it (within the 186-day window). Let the corrected sample be ${\widetilde{X}}_{i,\alpha }$ and ${\widetilde{{\rm{mean}}}}_{\alpha }$ be the mean of the corrected samples in group α. We use the following testing statistics:

$${\rm{AUC}}=\frac{{\sum }_{\alpha }{\widetilde{{\rm{mean}}}}_{\alpha }}{\widetilde{{\rm{std}}}}$$

where $\widetilde{{\rm{std}}}$ is calculated by keeping track of the weights for each samples so that AUC~*N*(0, 1) under the null hypothesis. Then the *P* values can be calculated accordingly.

Moreover, we compared the AUC performance with one of the standard methods for longitudinal analysis, that is, linear regression (LR) analysis with the time covariate. We used the standard LR analysis using the python implementation (statsmodel.OLS)^65^. We used time as a real valued covariate and the individual ID as a categorical covariate. Both AUC and LR methods performed well in identifying differentially expressed molecules (Extended Data Fig. 4a). While more than half of features identified by the LR method as differentially expressed molecules were also found by the AUC method (that is, 53% for RNA-seq), the AUC method identified more features than the LR method (Extended Data Fig. 4a). We therefore used the AUC test for our differential expression/abundance analyses over the stress events.

In order to assess differential changes per each event category (that is, only EE versus personal baseline), we used the paired *t*-test. The choice of paired *t*-test was to standardize the analysis across all different types of ’omics data. We performed the AUC test and paired *t*-test on pre-processed data, including transcriptome, metabolome, proteome, cytokine, r16S gut microbiome, r16S nasal microbiome, microbial predicted genes (KO genes by KEGG), and clinical laboratory test data. Transcriptome data were normalized according to size factor and converted into the log space by log(*X*_*ij*_ + 0.5) for downstream analyses. For size factor correction, first the geometric mean of each gene expression across all samples was calculated. The size factor for each sample is the median across genes of the ratio of the expression to the gene’s geometric mean. Then the read counts for each sample were normalized by the size factor. The size factor correction was carried out as described in DESeq2^55,66^.

We used *q*-values for false discovery rate control. We considered molecules with *q* < 0.1 to be significant. It is of note that for stage pairwise comparisons (for example, EE versus personal healthy baseline), we compared the performance of the paired *t*-test with the DESeq2 method for differential analyses of transcriptome data (Extended Data Fig. 4b, Supplementary Table 39). The number of differentially expressed transcripts (*q* < 0.1) identified by paired *t*-test was 6,857 and the number of differentially expressed transcripts (*q* < 0.1) identified by the DESeq2 method was 4,062, of which 71% overlapped with paired *t*-test results (Extended Data Fig. 4b). Moreover, pathway enrichment analyses for differentially expressed transcripts by both methods showed the same pathway enrichment results (Supplementary Table 40). Because the difference between results obtained from the paired *t*-test and Deseq2 methods for transcripts was minor, we decided to use the paired *t*-test for our analyses in order to standardize the analysis across all different types of ’omics data.

We applied ingenuity pathway analysis (IPA)^67^ to search for enriched pathways in our list of differentially expressed ’omics molecules. For integrated canonical pathway analysis, the significant transcripts, proteins, metabolites and cytokines were combined and used as an input file along as their respective *P* values and AUC statistics. The AUC statistic is used by IPA for generating pathway activity *z*-scores to predict activation or inhibition of enriched pathways. For event category analysis (that is, only EE versus personal baseline), we used paired *t*-test *P* values of significant ’omics molecules and log_2_(baseline normalized read counts) for changes in expression or abundance as input for IPA analyses. The IPA enrichment algorithm uses two scores that addresses two independent aspects of the analyses. The first is the ‘enrichment’ score based on Fisher’s exact test *P* value. The *P* value represents the significance of the overlap between observed and predicted regulated molecules. The second score is the activation *Z*-score, which is a prediction measurement for the activation or inhibition state of the regulators in the pathways. Please note that the activation *Z*-score value of zero for pathways that have significant *P* values means that the IPA algorithm could not predict the activation or inhibition of the pathway and regulators^67^.

Furthermore, in order to uncover trends in statistically significant (AUC test *q* < 0.1) ’omics responses to stress events, we used longitudinal pattern recognition using fuzzy *c*-means^68^ clustering across all the data. We first used the elbow method to identify the optimal number of clusters in our data set. Data from the transcriptome, proteome, metabolome, cytokines, clinical laboratory tests and 16S gut and 16S nasal microbiomes, as well as microbial KO genes, were standardized to *Z*-scores for each analyte and subjected to *c*-means clustering over the course of RVI or immunization. Each subplot in Extended Data Figs. 5, 6 shows a unique cluster and is colour coded on the basis of correlation membership scores. The top integrated canonical pathways (transcripts, proteins, metabolites, cytokines), and the trends for the top other analytes (microbiome and clinical laboratory tests) are shown above each plot.

### Classification of stress events

In order to predict stress events (that is, RVI versus healthy time points), we tested multiple models, of which the LR and the SVM performed best. Two predictions were executed: 1) healthy baselines versus RVI (Fig. 4c, Extended Data Fig. 8), and 2) healthy baselines versus immunization (Extended Data Fig. 9).

#### Data preparation

We used eight sets of data: transcriptome, cytokines, metabolome, proteome, r16S gut microbiome, r16S nasal microbiome, PBMC deconvoluted cell types, and clinical laboratory test data. For the transcriptome data, we applied VST (variance stabilization transformation) from the DeSeq2 algorithm^55^ and used a subset of genes that are immune-related based on previous studies^56^ (Supplementary Table 27). For cytokines, metabolome, proteome and r16S data, we corrected for the size factor. Features with more than 100 missing values were discarded. Also, healthy time points with HS-CRP values larger than 10 were discarded. The feature HS-CRP was not used for prediction, as we already used this information to filter out samples. Finally, we applied *Z*-transformation to all features so that they had means of 0 and variances of 1 (across the time points).

We executed LR and SVM for our prediction models, as implemented in python package sklearn. For both methods, l1 regularization is used to encourage the sparsity of the learned coefficient. Two prediction experiments were performed: healthy versus RVI, and healthy versus immunization. We only used the infection and immunization time points close to the onset of RVI (EE and EL groups). The prediction performance was evaluated by receiver operating characteristic (ROC) curves and areas under the ROC curve (AUC) for every ’ome (transcriptome, metabolome, proteome, cytokines, PBMC cells types, r16S gut microbial taxa, r16S nasal microbial taxa and clinical laboratory tests) and also of all ’omes combined (or multi-omes). The ROC plot shows true positive rate (TPR) against false positive rate (FPR). The curve was calculated by varying the decision threshold to have different TPR–FPR tradeoffs.

For every experiment, we randomly selected 70% of data for the training set and 30% of data for the testing set. Time points from the same individual were used in only one of the two sets. This was repeated 100 times. The regularization parameters were selected based solely on the training set, as follows. For each regularization parameter *C* over the set [0.1, 0.5, 1, 2, 3, 5, 10], the training data were further split into train_train and train_test. A classification model (LR or SVM) was trained on train_train and evaluated on train_test. This was done five times and the regularization parameter with the smallest error on train_test was chosen as the optimal parameter. Then, the model was trained with the entire training set and the optimal regularization parameter, and evaluated on the testing set.

### Correlation network analysis

Given the Simpson’s paradox in correlational analyses (wherein trends can disappear or reverse when data sets are combined)^69^, we used two statistical approaches to investigate between-individual and within-individual correlations separately, as these reveal different perspectives in understanding the associations.

#### Within-individual correlations (at the personal level)

This takes all the healthy visits per subject into account, and used linear mixed effect models to account for repeated samplings from the same subject. We used the rmcorr method^64^, which is close to a null multilevel model of varying intercept and a common slope for each individual, and specifically tests for a common association between variables within each subject. Healthy visits were first grouped into insulin-sensitive and insulin-resistant, and each analyte was linearly transformed before applying the rmcorr() function from the rmcorr R package as explained above (see ‘Associations with time’). As this method relies on repeated measures within each subject and specifically tests for a common association between variables within each subject, potentially confounding factors between subjects, such as sex, age and BMI, do not apply, which is in contrast to the between-individual correlation method below.

#### Between-individual correlations (at the cohort level)

This first takes the median value of all healthy visits per subject, linearly transforms and then corrects for sex, age and BMI before applying the regression pcor.test() function from the ppcor R package. As this method replies on the median value of repeated measures within each subject, those become independent observations presenting different subjects, so this is suitable for standard linear regression methods downstream.

*P* values obtained from the above two approaches were further multiple hypothesis corrected by the total number of pairwise comparisons using the FDR method as implemented by p.adjust(p.value, method = “fdr”) in R. We used *q* < 0.05 as the significant cutoff for all ’omic analytes.

For microbiome-related networks, we implemented two approaches to construct a correlational network that accounts for the compositionality effect. In the first approach, we used centered log ratio (CLR)^70^ as a preprocessing transformation method that addresses compositionality in microbial data^71^. Given a sample with *D* taxa, the CLR transformation can be obtained as follows:

$$\begin{array}{l}{x}_{{\rm{CLR}}}=\left[{\rm{log}}\left({x}_{1}/G\left(x\right)\right),{\rm{log}}\left({x}_{2}/G\left(x\right),....,{\rm{log}}\left({x}_{D}/G\left(x\right)\right)\right)\right],\\ G\left(x\right)=\sqrt[D]{{x}_{1}{x}_{2}{x}_{3}...{x}_{D}}\end{array}$$

Because microbial taxa span different taxonomic levels (phylum, class, order, family, genus), we used CLR transformation on each taxonomic level separately. After accounting for compositional effect via CLR transformation, we addressed the intra-personal correlation of repeated measurements in the calculation of correlation coefficient between taxa by using repeated measures correlation (rmcorr) method^64^. Hence, by using this approach, we accounted for compositional effects via CLR and repeated measurements via rmcorr (CLR + rmcorr). For all microbiome–host networks (Fig. 5c, d), we used this approach to calculate the correlation to host ’omics (transcriptomics, proteomics, metabolomics, cytokines, and clinical data).

In the second approach, we used SparCC^72^ to construct a microbial–microbial network over repeated measurements^73^ (Fig. 5a, b). We used the python implementation of SparCC in https://bitbucket.org/yonatanf/sparcc with parameters: –iter = 20,–xiter = 10,–threshold = 0.1. Also, we obtained the *P* value for each correlation coefficient by bootstrapping the data set 100 times and applying SparCC to each of those 100 data sets. We applied SparCC to features from each taxonomic rank separately to avoid correlations between parent and child taxa. We further compared the microbiome–microbiome network calculated either by SparCC or by CLR+rmcorr (Supplementary Table 32). Correlation coefficients obtained by either method were linearly associated, and more than 50% of significant correlations overlapped, indicating that both methods generally agree. However, some correlations were detected by only one method. Future studies are needed to improve the microbial correlation techniques, as also suggested previously^73^.

### Outlier analysis

To account for healthy baseline variability, only subjects with at least three healthy visits were included in the analysis. *Z*-scores were calculated for each analyte after log_2_-transformation using the median value among healthy visits for each subject. Outliers were defined as being in the 95th percentile of *Z*-score distribution for each analyte. The outlier proportion across assays was calculated by normalizing the number of outliers from each assay to the total number of analytes profiled with the corresponding assay. The percentage of outlier analytes across assays in each participant was then normalized to 100%. Analytes with more than 50% of missing values or zeros were discarded. For transcriptomics data, we arbitrarily chose to discard genes with low expression (log_2_ normalized read count <5 in more than 50% of the subjects).

While providing tremendous amounts of molecular information, our study has several limitations that necessitate future investigation. First, it is possible that protein isoforms and/or transcript variants are also critical for accurately evaluating host states, which were not examined in the current study. Our studies of microbial changes are also limited, as microbial profiles are based on 16S sequencing, which only allows taxonomy assignments at the genus level and thereby limits more precise interpretations requiring species or strain classifications. Second, as our study is observational, samplings in our study are both planned (most healthy visits) and spontaneous (some healthy visits and most stress visits), resulting in uneven collections. In addition, we utilized a variety of techniques to profile different molecules, so each type of data set has its inherent errors specific to its corresponding platforms. As such, our data are heterogenous by nature, and require custom and novel methods that statistically account for many sources of variation. Our analyses here tend to account for some sources of variation, but additional methods are necessary for future work. Last, we only considered BMI, age and sex as the universal confounding factors in the current study for our correlational analyses. However, there may be additional factors, such as diet and exercise, that need to be considered. For instance, in our analysis of age associations, we cannot exclude the possible influence of changes in lifestyle over the profiling period. Nonetheless, as our cohort is currently expanding to include more participants with continuing longitudinal samplings and archived biobanked collections, we believe it will provide a rich and valuable resource for future research both experimentally and informatically.

### Reporting summary

Further information on research design is available in the Nature Research Reporting Summary linked to this paper.

## Online content

Any methods, additional references, Nature Research reporting summaries, source data, statements of data availability and associated accession codes are available at 10.1038/s41586-019-1236-x.

## Supplementary information

Supplementary Information This file contains a full guide for Supplementary Tables 1-40.

Reporting Summary

Supplementary Tables This file contains Supplementary Tables 1-29 and 32-40 – see Supplementary Information document for full descriptions.

Supplementary Table 30 Multi-omic within-individual correlations among molecules in each ome (except microbiome to microbiome) at healthy baselines (IR=215, IS=238, all=624 including baselines from IR/IS undetermined individuals). Pearson correlation coefficient r, the p-value, 95% confidence intervals (CI) and FDR corrected p-values are listed. IS associations are listed first followed by IR associations. Significant associations (FDR 5%) at either group are listed. The p-value for linear mixed model and its interaction term to test IR/IS difference was also listed for each comparison.

Supplementary Table 31 Multi-omic between-individual correlations among molecules in each ome (except microbiome to microbiome) and between host and gut microbial profiles at healthy baselines (IR=215, IS=238, all=624 including baselines from IR/IS undetermined individuals). Pearson correlation coefficient r, the p-value and FDR corrected p-values are listed. Associations using all subjects are listed first, followed by associations in IS subjects and then those in IR subjects. Significant associations (FDR 5%) at either group are listed.

## Data Availability

Raw data included in this study are hosted on the NIH Human Microbiome 2 project site (https://portal.hmpdacc.org) with no restrictions on its use. Exome sequencing data are also available at dbGaP under Study Accession phs001719.v1.p1. Both raw and processed data are also hosted on the Stanford iPOP site (http://med.stanford.edu/ipop.html).

## Code availability

Available data and tools were developed as open source, with CC0 for data and MIT for software as open-source licenses. Processed data and custom analysis scripts are hosted on the Stanford iPOP site (http://med.stanford.edu/ipop.html).
