# Supplementary material for: Longitudinal multi-omics of host–microbe dynamics in prediabetes
Source: Nature. 2019 May 29;569(7758):663–71. doi: 10.1038/s41586-019-1236-x (PMC6666404; doi:10.1038/s41586-019-1236-x)
Supplement: Supplementary file 1 — This file contains a full guide for Supplementary Tables 1-40. [file 41586_2019_1236_MOESM1_ESM.pdf]

nature
SUPPLEMENTARY INFORMATION
https://doi.org/10.1038/s41586-019-1236-x
In the format provided by the authors and unedited.

Longitudinal multi-omics of host–microbe dynamics in prediabetes

Wenyu Zhou[1,12], M. Reza Sailani[1,12], Kévin Contrepois[1,12], Yanjiao Zhou[2,3,12], Sara Ahadi[1,12], Shana R. Leopold[2], Martin J. Zhang[4], Varsha Rao[1], Monika Avina[1], Tejaswini Mishra[1], Jethro Johnson[2], Brittany Lee-McMullen[1], Songjie Chen[1], Ahmed A. Metwally[1], Thi Dong Binh Tran[2], Hoan Nguyen[2], Xin Zhou[2], Brandon Albright[2], Bo-Young Hong[2], Lauren Petersen[2], Eddy Bautista[2], Blake Hanson[2], Lei Chen[2], Daniel Spakowicz[2], Amir Bahmani[5], Denis Salins[1], Benjamin Leopold[2], Melanie Ashland[1], Orit Dagan-Rosenfeld[1], Shannon Rego[1], Patricia Limcaoco[1], Elizabeth Colbert[6], Candice Allister[6], Dalia Perelman[6], Colleen Craig[6], Eric Wei[1,5], Hassan Chaib[1,5,7], Daniel Hornburg[1], Jessilyn Dunn[1], Liang Liang[1], Sophia Miryam Schüssler-Fiorenza Rose[8,9], Kim Kukurba[1], Brian Piening[10], Hannes Rost[11], David Tse[4], Tracey McLaughlin[6,7], Erica Sodergren[2], George M. Weinstock[2]* & Michael Snyder[1,5,7]*

[1]Department of Genetics, Stanford University School of Medicine, Stanford, CA, USA. [2]The Jackson Laboratory for Genomic Medicine, Farmington, CT, USA. [3]Department of Medicine, UConn Health, Farmington, CT, USA. [4]Department of Electrical Engineering, Stanford University, Stanford, CA, USA. [5]Stanford Center for Genomics and Personalized Medicine, Stanford, CA, USA. [6]Division of Endocrinology, Stanford University School of Medicine, Stanford, CA, USA. [7]Stanford Diabetes Research Center, Stanford, CA, USA. [8]Spinal Cord Injury Service, Veteran Affairs Palo Alto Health Care System, Palo Alto, CA, USA. [9]Department of Neurosurgery, Stanford University School of Medicine, Stanford, CA, USA. [10]Earle A Chiles Research Institute, Providence Cancer Center, Portland, OR, USA. [11]Donnelly Centre for Cellular & Biomolecular Research, University of Toronto, Toronto, Ontario, Canada. [12]These authors contributed equally: Wenyu Zhou, M. Reza Sailani, Kévin Contrepois, Yanjiao Zhou, Sara Ahadi. *e-mail: George.Weinstock@jax.org; mpsnyder@stanford.edu

Supplementary Information Tables

Supplementary Information Table 1: Clinical characters of the cohort, including SSPG, sex, ethnicity, age, BMI and number of visits (All and only healthy baselines) and time span in the study. Column H Class provides a classification of individuals based on their longitudinal A1C, fasting glucose and OGTT during our study.

Supplementary Information Table 2: Annual OGTT measurements in subjects of the cohort, with the date of test, the baseline fasting plasma glucose (FPG) and 2 hour glucose measurements listed.

Supplementary Information Table 3: List of 1092 samples in the study, with annotations on their days since the study start date (03/01/2013), physiological states (CL1-CL4), and whether profiled by multi-omic assays (0 for not-profiled, 1 for profiled).

Supplementary Information Table 4: List of multi-omic molecules analyzed with their interquartile range (IQR) across the cohort, the mean expression/abundance followed by IQRs in each individual. Additionally, the mean and standard deviation of individual IQR were listed, with the outlier individuals counted and listed.

Supplementary Information Table 5: List of molecules with their variance decomposition of healthy visits across the cohort to fixed effects (Days, A1C, SSPG and FPG), random effect (SubjectID) and intra-class correlation (ICC). The total variance was normalized to one for each molecule before applying linear mixed effect models to allow molecules cross-comparable to each other.

Supplementary Information Table 6: The median and mean distance score between all individuals (Ind_score, n=106) when top n personally variable molecules selected, followed by the percentages of those n molecules that are from different omes.

Supplementary Information Table 7: List of example molecules that were significantly associated with the time factor among the healthy baselines, with their Pearson r value (cor_r), un-corrected p-value (cor_p), 95% confidence intervals (CI_left and CI_right) and FDR multiple hypothesis corrected q-values (p.adj). Both all individuals (All, n=106) and those 27 who had more than 900 days' collections (D900) were included in the correlation analyses to compare.

Supplementary Information Table 8: Matrix listing molecules that were significantly associated with SSPG values between subjects in the cohort. For each molecule (row), the expression/abundance value was listed as the median of healthy baselines per subject (column). The status was listed as "YES" if the molecule also significantly associated with IR/IS classification shown in Supplemental Table 9. Both FDR p-values and Pearson r were listed afterwards. Additionally, correlation p values were listed for potential confounding factors such as HDL, triglycerides (TGL), triglycerides/HDL ratio, statin intake and glucose control medications (GLC).

Supplementary Information Table 9: Matrix listing molecules that significantly differed between IR and IS subjects. For each molecule (row), the expression/abundance value was listed as the median of healthy baselines per subject (column). FDR p-values were listed afterwards.

Supplementary Information Table 10: List of molecules from all multi-omic measurements that significantly deviated from the personal baseline over the course of respiratory viral infection (RVI) according to AUC test (two-sided, q-value < 0.1) based on 156 RVI and 89 healthy categorized time point. The nominal p values and AUC statistics are listed per omics molecule/measurement.

Supplementary Information Table 11: List of the top 100 enriched pathways (two-sided Fisher's exact test) integrating cytokines, transcripts, proteins and metabolites that were differentially changed in response to RVI (156 RVI and 89 healthy categorized time point).

Supplementary Information Table 12: List of differentially expressed omics molecules with their membership scores (or correlation coefficient) for four clustering patterns during RVI. The patterns were shown as in Extended Data Figure 5a.

Supplementary Information Table 13: List of molecules from all multi-omic measurements that significantly deviate from the personal baseline over the course of RVI in the IR group (n=33 RVI and n=17 healthy categorized time points) according to AUC test (two-sided). The nominal p values, AUC statistics are listed per omics molecule/measurement.

Supplementary Information Table 14: List of molecules from all multi-omic measurements that significantly deviate from the personal baseline over the course of RVI in the IS group (n=77 RVI and n=62 healthy categorized time points) according to AUC test (two-sided). The nominal p values, AUC statistics are listed per omics molecule/measurement.

Supplementary Information Table 15: List of the top 100 enriched pathways integrating cytokines, transcripts, proteins and metabolites that were differentially changed in response to RVI in IR group (n=33 RVI and n=17 healthy categorized time points) based on two-sided Fisher`s exact test as implemented in ingenuity IPA program.

Supplementary Information Table 16: List of the top 100 enriched pathways integrating cytokines, transcripts, proteins and metabolites that were differentially changed in response to RVI in IS group (n=77 RVI and n=62 healthy time points) based on two-sided Fisher`s exact test as implemented in ingenuity IPA program.

Supplementary Information Table 17-19: List of molecules from cytokines, metabolites and transcripts that significantly changed during RVI in pairwise comparison of event early (EE, n=67, SI Table 17), event late (EL, n=28, SI Table 18) and recovery early (RE, n=61, SI Table 19) to personal healthy baselines based on two-sided paired-t test, respectively. Fold change was calculated as ratio of the expression/abundance of molecules in each category in regard to personal baseline. Q values were also listed per molecules.

Supplementary Information Table 20: List of molecules from all multi-omic measurements that significantly deviate from the personal baseline over the course of immunization (117 immunization and 59 healthy categorized time points) according to AUC test (two-sided). The nominal p values, AUC statistics are listed per omics molecule/measurement.

Supplementary Information Table 21: List of the top 100 enriched pathways based on two sided Fisher`s exact test as implemented in ingenuity IPA program, that integrate cytokines, transcripts and metabolites that were differentially changed in response to immunization (n=117).

Supplementary Information Table 22: List of molecules with their membership scores (or correlation coefficient) for four clustering patterns in response to immunization (n=117). The patterns were shown as in Extended Data Figure 6a.

Supplementary Information Table 23-25: List of molecules from cytokines, metabolites and transcripts that significantly changed during immunization in pairwise comparison of event early (EE, n=59, SI Table 23), event late (EL, n=29, SI Table 24) and recovery early (RE, n=29, SI Table 25) to personal healthy baselines based on Fisher's exact test as implemented in ingenuity IPA program, respectively. Fold change was calculated as ratio of the expression/abundance of molecules in each category in regard to personal baseline. Q values were also listed per molecules.

Supplementary Information Table 26: List of immune molecules related to type II diabetes development and MODY genes and their respected P-values and AUC (two-sided) statistics during RVI (n=156) and immunization (n=117).

Supplementary Information Table 27: List of genes that are immune related and used for classification of stress events for transcriptome.

Supplementary Information Table 28: The top 100 omics features for classification of RVI selected by both LR and SVM models. The order is based on the sum of the coefficient value in LR and SVM. The coefficient value is calculated as an average of the absolute value of 100 repetitions and is a significance indicator of corresponding feature.

Supplementary Information Table 29: The top 100 omics features for classification of immunization selected by both LR and SVM models. The order is based on the sum of the coefficient value in LR and SVM. The coefficient value is calculated as an average of the absolute value of 100 repetitions and is a significance indicator of corresponding feature.

Supplementary Information Table 30: Multi-omic within-individual correlations among molecules in each ome (except microbiome to microbiome) at healthy baselines (IR=215, IS=238, all=624 including baselines from IR/IS undetermined individuals). Pearson correlation coefficient r, the p-value, 95% confidence intervals (CI) and FDR corrected p-values are listed. IS associations are listed first followed by IR associations. Significant associations (FDR 5%) at either group are listed. The p-value for linear

mixed model and its interaction term to test IR/IS difference was also listed for each comparison.

Supplementary Information Table 31: Multi-omic between-individual correlations among molecules in each ome (except microbiome to microbiome) and between host and gut microbial profiles at healthy baselines (IR=215, IS=238, all=624 including baselines from IR/IS undetermined individuals). Pearson correlation coefficient r, the p-value and FDR corrected p-values are listed. Associations using all subjects are listed first, followed by associations in IS subjects and then those in IR subjects. Significant associations (FDR 5%) at either group are listed.

Supplementary Information Table 32: List of within-individual associations among microbiome that were either IR or IS significant (IR=184, IS=190, as not all visit had stool sampling). Results both by SparCC method and by CLR+rmcorr method are listed. For results by SparCC method, correlation coefficient (rho) and p values are listed only for significant correlations either in IS/IR subjects. For results by CLR+rmcorr method, their Pearson r value (cor_r), un-corrected p-value (cor_p), 95% confidence intervals (CI_left and CI_right) and FDR multiple hypothesis corrected p-values (p.adj) were listed.

Supplementary Information Table 33: List of within-individual associations between host omes and gut microbes that were either IR or IS significant. For each association, its pearson r value (cor_r), un-corrected p-value (cor_p), 95% confidence intervals (CI_left and CI_right) and FDR multiple hypothesis corrected p-values (p.adj) were listed.

Supplementary Information Table 34: List of outlier molecules in each subject as compared to the cohort mean. For each outlier, its Z-score, FDR corrected p value and omic type are listed.

Supplementary Information Table 35: List of the significantly enriched toxicity pathways from outliers in subject ZJTKAE3.

Supplementary Information Table 36: Matrix lists the sum of absolute value of Z-scores across each omes with their ascending ranks (columns) for each sample (row).

Supplementary Information Table 37-38: Matrix lists molecules that were significantly associated with IL1RA (SI Table 37) or HSCRP (SI Table 38) in subject ZNED4XZ. For each molecule (row), the expression/abundance value was listed for each visit of this subject (column). Both FDR p-values and Pearson correlation r were listed afterwards.

Supplementary Information Table 39: List of differentially expressed transcripts ($q < 0.1$) identified by paired-t test (two-sided) or DESeq2 method (Wald test) for stage wise comparison (RVI EE versus personal healthy baselines).

Supplementary Information Table 40: Pathway enrichment analyses results for differentially expressed transcripts listed in SI Table 39 identified by paired t-test (two-sided) or DESeq2 method (Wald test).
