# Supplementary material for: Longitudinal multi-omics of host–microbe dynamics in prediabetes
Source: Nature. 2019 May 29;569(7758):663–71. doi: 10.1038/s41586-019-1236-x (PMC6666404; doi:10.1038/s41586-019-1236-x)
Supplement: Supplementary file 2 — Reporting Summary [file 41586_2019_1236_MOESM2_ESM.pdf]

nature research

Corresponding author(s): G. Weinstock and M. Snyder

Last updated by author(s): Apr 11, 2019

Reporting Summary

Nature Research wishes to improve the reproducibility of the work that we publish. This form provides structure for consistency and transparency in reporting. For further information on Nature Research policies, see Authors & Referees and the Editorial Policy Checklist.

Statistics

For all statistical analyses, confirm that the following items are present in the figure legend, table legend, main text, or Methods section.

n/a	Confirmed	
☐	☒	The exact sample size (*n*) for each experimental group/condition, given as a discrete number and unit of measurement
☐	☒	A statement on whether measurements were taken from distinct samples or whether the same sample was measured repeatedly
☐	☒	The statistical test(s) used AND whether they are one- or two-sided<br>*Only common tests should be described solely by name; describe more complex techniques in the Methods section.*
☐	☒	A description of all covariates tested
☐	☒	A description of any assumptions or corrections, such as tests of normality and adjustment for multiple comparisons
☐	☒	A full description of the statistical parameters including central tendency (e.g. means) or other basic estimates (e.g. regression coefficient) AND variation (e.g. standard deviation) or associated estimates of uncertainty (e.g. confidence intervals)
☐	☒	For null hypothesis testing, the test statistic (e.g. *F*, *t*, *r*) with confidence intervals, effect sizes, degrees of freedom and *P* value noted<br>*Give P values as exact values whenever suitable.*
☒	☐	For Bayesian analysis, information on the choice of priors and Markov chain Monte Carlo settings
☐	☒	For hierarchical and complex designs, identification of the appropriate level for tests and full reporting of outcomes
☐	☒	Estimates of effect sizes (e.g. Cohen's *d*, Pearson's *r*), indicating how they were calculated

Our web collection on statistics for biologists contains articles on many of the points above.

Software and code

Policy information about availability of computer code

Data collection	Progenesis QI for metabolomics data, pyProphet for proteomics data
Data analysis	Open-sourced and standard packages include: TopHat, HTseq, DESeq2, Uchime,<br>Usearch, RDP-classifer, QIIME, TRIC, Perseus.

For manuscripts utilizing custom algorithms or software that are central to the research but not yet described in published literature, software must be made available to editors/reviewers. We strongly encourage code deposition in a community repository (e.g. GitHub). See the Nature Research guidelines for submitting code & software for further information.

Data

Policy information about availability of data

All manuscripts must include a data availability statement. This statement should provide the following information, where applicable:

- Accession codes, unique identifiers, or web links for publicly available datasets
- A list of figures that have associated raw data
- A description of any restrictions on data availability

Raw data included in this study are hosted on the NIH Human Microbiome 2 project site (https://portal.hmpdacc.org) with no restrictions for its use. Exome sequencing data are also available at dbGaP under Study Accession phs001719.v1.p1.

Field-specific reporting

Please select the one below that is the best fit for your research. If you are not sure, read the appropriate sections before making your selection.

☒ Life sciences ☐ Behavioural & social sciences ☐ Ecological, evolutionary & environmental sciences

For a reference copy of the document with all sections, see nature.com/documents/nr-reporting-summary-flat.pdf

Life sciences study design

All studies must disclose on these points even when the disclosure is negative.

Sample size	Sample size for the main HMP project was calculated based on the pilot iPOP study findings of levels of autocorrelation in multiomics data and the number of timepoints needed per participant to reconstruct the time series with < 1% uncertainty. Additionally, our observational study is different from traditional cross-section study in two aspects: 1) We sample more than one time longitudinally in each of the participants; 2) We focus on naturally or spontaneously occurring physiological states. For group-wise comparison, we examined on those had at least 30 individuals or events per group (IR vs IS, Infection vs healthy, Immunization vs infection etc) and used statistical analyses to take the sample size into account. For our analyses focusing on individual characters, we examined on those participants who had at least three independent samplings in our study. The sample size is confirmed sufficient by using a stringent significant threshold in downstream statistics.
Data exclusions	For microbiome analyses, to capture major changes across the whole cohort, we focused on prevalent taxa and genes out of all mapped ones, excluding those that were present in less than half of the cohort.
Replication	To control data generation reproducibility, we included multiple aliquots for selected samples anonymously in different batches of all omic assays, and make sure replicates were similar to each other than to any other samples.
Randomization	Samples were randomly arranged by computing program into various batches of omics assay.
Blinding	Each sample was labeled with a randomized ID whose annotation and clinical status were kept blinded during data collection and analyses.

Reporting for specific materials, systems and methods

We require information from authors about some types of materials, experimental systems and methods used in many studies. Here, indicate whether each material, system or method listed is relevant to your study. If you are not sure if a list item applies to your research, read the appropriate section before selecting a response.

Materials & experimental systems

n/a	Involved in the study	
☒	☐	Antibodies
☒	☐	Eukaryotic cell lines
☒	☐	Palaeontology
☒	☐	Animals and other organisms
☐	☒	Human research participants
☒	☐	Clinical data

Methods

n/a	Involved in the study	
☒	☐	ChIP-seq
☒	☐	Flow cytometry
☒	☐	MRI-based neuroimaging

Human research participants

Policy information about studies involving human research participants

Population characteristics	55 participants in this study were female and 51 male, with ages ranging from 25 to 75 yrs old and BMI 25-40 kg/m2. More information were provided in Methods and Supplemental Table 1.
Recruitment	Participants were recruited via placement of advertisements in local newspapers and radio stations seeking “prediabetic volunteers” at risk for Type 2 diabetes for longitudinal multi-omic study. Screening in the CTRU entailed history and physical, anthropometric measurements, and fasting blood tests for exclusions including presence of anemia defined as hematocrit < 30, renal disease defined as creatinine > 1.5, history of any cardiovascular, malignancy, chronic inflammatory, psychiatric disease, and history of any bariatric surgery or liposuction.
Ethics oversight	Stanford University Institutional Review Board (IRB 23602)

Note that full information on the approval of the study protocol must also be provided in the manuscript.
